# Clinical Strains of *Helicobacter pylori* With Strong Cell Invasiveness and the Protective Effect of Patchouli Alcohol by Improving miR-30b/C Mediated Xenophagy

**DOI:** 10.3389/fphar.2021.666903

**Published:** 2021-04-30

**Authors:** Yifei Xu, Qiuhua Deng, Yuanzun Zhong, Li Jing, Haiwen Li, Jingwei Li, Huimin Yu, Huafeng Pan, Shaoju Guo, Hongying Cao, Ping Huang, Bin Huang

**Affiliations:** ^1^ Shenzhen Traditional Chinese Medicine Hospital, the Fourth Clinical Medical College of Guangzhou University of Chinese Medicine, Shenzhen, China; ^2^ School of Pharmaceutical Sciences, Guangzhou University of Chinese Medicine, Guangzhou, China; ^3^ School of Basic Medical Science, Tianjin Medical University, Tianjin, China; ^4^ School of Medicine, Shenzhen University, Shenzhen, China; ^5^ Science and Technology Innovation Center, Guangzhou University of Chinese Medicine, Guangzhou, China

**Keywords:** helicobacer *pylori*, patchouli alcohol, xenophagy, Intracellular, drug resistance, miR-30

## Abstract

*Helicobacter pylori* was classified by the World Health Organization as a class 1 carcinogen. The development of drug-resistant strains of this pathogen poses a serious threat to human health worldwide. The cell invasion of *H. pylori* activates xenophagy in gastric epithelial cells by mediating miR-30b/c, and the emergence of autophagosomes provides a niche that enables the survival of intracellular *H. pylori* and promotes its drug resistance. This study revealed that some clinical drug-resistant *H. pylori* strains present much stronger invasive ability than standard strains. Patchouli alcohol (PA), a tricyclic sesquiterpene from *Pogostemon cablin* (Blanco) Benth (Labiatae), showed reliable activity against intracellular *H. pylori*. The mechanisms appeared to involve the downregulation of miR-30c-3p/5p and miR-30b-5p, thereby upregulating xenophagy-related gene expression (ULK1, ATG5, ATG12, and ATG14) and enhancing xenophagy. PA also inhibited the nuclear transfection of miR-30b-5p induced by *H. pylori*, thereby enhancing transcription factor EB function and increasing lysosome activity. The finding of strongly invasive intracellular *H. pylori* has great implications for clinical treatment, and PA can act against invasive *H. pylori* based on the improvement of miR-30b/c mediated xenophagy. Taken together, the results demonstrate that PA have potential use as a candidate medication for intracellular drug-resistant *H. pylori*.

## Introduction

*Helicobacter pylori* was classified by the World Health Organization as a class 1 carcinogen, infecting half of the world’s population (Ferrero et al., 2012). *H. pylori* plays a major role in gastric ulcer, atrophic gastritis, and gastric carcinoma. Triple therapy (a proton pump inhibitor combined with two antibiotics, like clarithromycin [CLR], metronidazole [MTZ]) are adopted to treat the infection, however, the increasing drug-resistant rate of *H. pylori* definitely undermine the efficacy of eradication treatment. The infection process of *H. pylori* is fairly complex. First, the bacteria penetrate the gastric mucosa via its flagella and chemotaxis protein (Lertsethtakarn et al., 2011). It then adheres to the surface of gastric epithelial cells; some bacteria can invade and survive inside epithelial cells. The ability of *H. pylori* to survive intracellularly is believed to be one of the strategies through which it escapes the action of drugs (Necchi et al., 2007). As the bacterium invades host gastric epithelial cells, the tolerance against drugs *H. pylori* is significantly enhanced (Chu et al., 2010).

When *H. pylori* invades epithelial cells, the immune response of the cells is activated. Xenophagy is a key protective response against bacterial invasion (Zhang et al., 2014). Autophagosomes wrap around intracellular bacteria and then merge with lysosomes to form autolysosomes; the bacteria are digested and eliminated thereafter (Sharma et al., 2018). However, over the course of its evolution, *H. pylori* has developed the ability to escape from autophagosomes. Vacuolating cytotoxin A (VacA), a toxic factor secreted by *H. pylori*, disrupts the initiation and digestion processes of xenophagy, which leads to the survival of *H. pylori* inside autophagosomes (Greenfield and Jones, 2013). Compromised xenophagy by miR-30b is considered to be one of the major mechanisms for the self-protective abilities of *H. pylori* (Tang et al., 2012). Thus, promoting xenophagy can enhance the elimination of intracellular bacteria (Liao et al., 2016; Sui et al., 2017).

**FIGURE 1:**
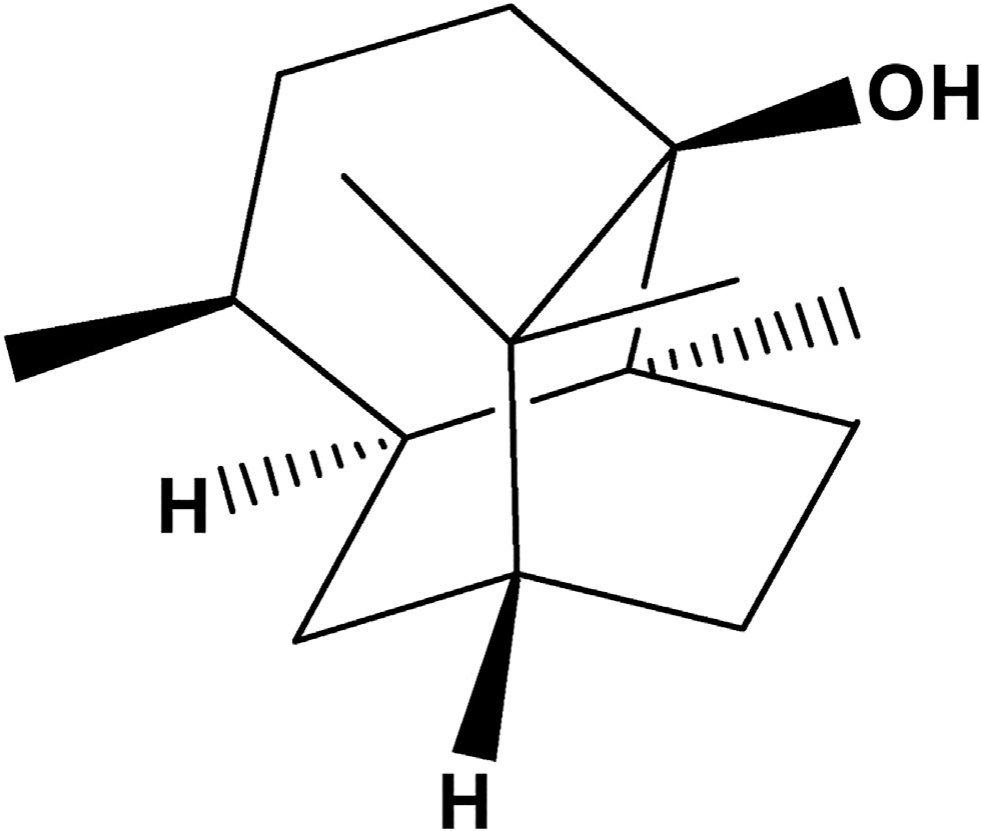
Chemical structure of PA.

Patchouli alcohol (Figure 1, PubChem CID: 10955174) is one of the major ingredients of *Pogostemon cablin* (Blanco) Benth (Labiatae), which is widely used to treat gastrointestinal diseases in East Asian countries. The effects of PA against *H. pylori* infection and induced gastritis *in vitro* and *in vivo* were demonstrated in our previous research (Xu et al., 2017; Lian et al., 2018). The inhibitory effect of PA on *H. pylori* urease highlights its reliable promotive effects on macrophage-mediated *H. pylori* digestion (Lian et al., 2019). In this study, we compared the cell invasive ability of some clinical drug-resistant and standard *H. pylori* strains, and investigated the effects of PA against intracellular *H. pylori* and the underlying mechanisms.

## Material and Methods

### Chemicals and Drugs

DMEM–high-glucose medium and fetal bovine serum (FBS) were purchased from Gibco (United States). Brain heart infusion (BHI) and Columbia agar base were obtained from OXOID (United States). Sheep blood was procured from Nanjing Maojie Biotechnology (China). Saponins were purchased from Sigma–Aldrich (United States). Gentamicin sulfate was purchased from Toku-E (Japan). CytoTrace™ Green CMFDA was obtained from AAT Bioquest (United States). mRFP-LC3-GFP adenovirus was purchased from HANBIO (China).

PA (purity >99%) was provided by the Mathematical Engineering Academy of Chinese Medicine, and its structure was confirmed as previously described (Qing et al., 2014). The sesquiterpene was dissolved in DMSO and adjusted to the necessary concentration for *in vitro* assays. PA solid dispersions were prepared using Poloxamer 407 as described in a previous publication for *in vivo* assays (Liao et al., 2015).

### *H. pylori* and Cell Culture

*H. pylori* strains NCTC11637 and NCTC26695 were purchased from the American Type Culture Collection. Sydney strain 1 (SS1) was gifted by Richard Ferrero from Monash University, Australia. The clinical strains Hp 1868, Hp 1869 (MTZ resistant strain), Hp 1870 (CLR and MTZ resistant strain), and Hp 1872 (MTZ resistant strain) were obtained from Renji Hospital, Shanghai Jiaotong University (The virulence genotypes of those *H. pylori* strains are shown in Supplementary Table S3). The identification and drug resistance of those strains was shown in our previous publication (Xu et al., 2017). *H. pylori* was cultured in Columbia agar base with 5% sheep blood at 37°C in a tri-gas incubator (Nuaire, United States) containing 10% CO_2_, 5% O_2_, and 85% N_2_, and the bacteria were passaged every 48 h. BHI supplemented with 30% glycerol was adopted as a cryoprotectant. The passage number of all strains were less than 20.

GES-1 and MKN45 cells were purchased from BNCC Biotechnology Company (China). GES-1 cells were cultured in DMEM–high-glucose medium (10% FBS), while MKN45 cells were cultured in RPMI 1640 (20% FBS). Both cell strains were cultured in an incubator at 37°C with 5% CO_2_. The cells were passaged using 0.25% trypsin when they covered over 80% of the dish. The passage number of those two cell lines were less than 20.

### Cells and Treatment

GES-1 or MKN45 cells were cultured in six-well plates at a density of 1.5×10^6^ cells/well. The cells were allowed to adhere to the walls of the wells for 12 h and then treated with 12.5, 25, or 50 μM PA or DMSO for 12 h. The doses of PA in *vitro* assay were selected based on our previous study (Lian et al., 2019). The cells were washed thrice with PBS. *H. pylori* was suspended in PBS, adjusted to appropriate quantity by a turbidity meter, and used to infect the cells at a multiplicity of infection (MOI) of 100:1. Rapamycin (100 nM, Meilune, China) and 3-MA (5 mM, Meilune, China) were selected as positive controls.

### Gentamycin Protection Assay

After drug pretreatment and *H. pylori* infection as described in “Cells and treatment” section, the cell culture media were collected, diluted, and cultured onto Columbia blood agar. The number of colonies formed 5 days later was counted as the extracellular bacterial number. The rest of the cells were washed twice with PBS and then incubated with 100 μg/ml gentamycin for 6 h to clear the extracellular bacteria. Subsequently, 0.1% saponin was adopted to increase membrane permeability, and the suspension was diluted and flooded on culture plates to form colonies. The number of colonies formed 5 days later was counted as the intracellular bacterial number.

### Lactate Dehydrogenase Release Measurement

GES-1 or MKN45 cells were cultured in six-well plates at a density of 1.5×10^6^ cells/well. The cells were divided into five groups, including the control (DMSO), *H. pylori* (DMSO + *H. pylori* for 12 h), and 12.5, 25, and 50 μM PA (pretreatment with the indicated dose of PA for 12 h and then infected with *H. pylori* at MOI = 100:1) treatment groups. After 4 h of co-culture (Supplementary Material S1), the supernatant was removed for lactate dehydrogenase (LDH) measurement (Nanjing Jiancheng Company, China).

### Immunofluorescence Assay and Confocal Microscopy

GES-1 cells were treated and infected with *H. pylori* for 12 h as described in “Cells and treatment” section. After treatment with 100 μg/ml gentamycin for 6 h, the adherent cells were washed thrice with PBS, fixed with 4% paraformaldehyde for 10 min, permeabilizated with 0.1% Triton-100 in PBST for 30 min, and blocked with 5% donkey serum in PBST for 1 h. The cells were then incubated with the primary antibodies, including anti-*H. pylori* (1:200, Abcam, ab20459, United States) and anti-transcription factor EB (TFEB, 1:500, Bethyl Laboratories, A303–673A, United States), dissolved in PBST containing 5% donkey serum at 4°C overnight. The next day, the samples were washed thrice with PBST and incubated with the secondary antibodies (1:200, goat anti-rabbit 488, Southern Biotech, 4,050–30, United States) for 2 h at room temperature. Hoechst 33,342 was used to stain the nucleus of the cells. The samples were mounted using Fluoromount-G™ (Invitrogen, United States) and observed by confocal microscopy (LSM 800, Zeiss, Germany).

### Transmission Electron Microscopy

GES-1 cells were treated and infected with *H. pylori* for 12 h as described in “Cells and treatment” section. After treatment with 100 μg/ml gentamycin for 6 h, the adherent cells were washed thrice with PBS and fixed with transmission electron microscopy (TEM) fixing solution (2.5% glutaraldehyde, LEAGENE, China). Cell scrapers was used to separate the cells, which were then collected by centrifugation at 1,000 rpm for 5 min. Finally, the samples were treated with 1 ml of fresh TEM fixing solution, followed by 1% osmium tetroxide, dehydrated with an acetone gradient, and embedded using Epon-812. After ultrathin sectioning, the samples were stained with uranyl acetate and lead citrate and observed by TEM.

### mRFP-LC3-GFP Adenovirus Transfection Assay

GES-1 cells were cultured in glass-bottomed dishes for 24 h and then added with 6.3 × 10^7^ PFU/ml mRFP-LC3-GFP adenovirus for 24 h of transfection (Supplementary Material S2, S3). The samples were divided into the following groups: ① without clear extracellular bacteria: DMSO (control), 200 nM rapamycin, 6.25 μg/ml *H. pylori* protein, *H. pylori* (MOI = 100:1), and 25 μM PA groups; ② with clear extracellular bacteria: DMSO (control), 100 μg/ml gentamycin (to exclude the possible influence of gentamycin on xenophagy), *H. pylori* (MOI = 100:1), and PA + *H. pylori* (pretreatment with 12.5, 25, or 50 μM PA for 12 h, infection with *H. pylori* at MOI = 100:1 for 12 h, and then treatment with 100 μg/ml gentamycin to clear extracellular bacteria) groups. *H. pylori* total protein extraction was followed by freeze–thawing cycling and sonification as described in our previous publication (Ren et al., 2019). Confocal microscopy was used to observe autophagosomes (yellow dots) and autolysosomes (red dots), and the numbers of autophagosomes and autolysosomes were analyzed using ImageJ.

### Lysosome Activity Assessment

LysoTracker Red DND-99 (Meilune, China) was used to identify the lysosome-rich areas of GES-1 cells. Briefly, GES-1 cells were cultured in glass-bottomed dishes, treated as described in “Cells and treatment” section, and then incubated in prewarmed 50 nM LysoTracker Red DND-99 for 1 h in a CO_2_ incubator. Finally, the staining liquid was replaced with fresh cell culture medium, and the samples were observed by confocal microscopy at 555 nm.

Cathepsin D activity was measured using a kit from Abcam (ab65302, United States). Briefly, GES-1 cells were treated as described in “Cells and treatment” section, washed twice with cold PBS, digested with 0.25% trypsin, and then resuspended in PBS. The cells were subsequently collected by centrifugation at 1,000 rpm and 4°C and resuspended in cell lysis buffer on ice for 10 min. The supernatants were obtained by centrifugation at 16,000× *g* for 5 min at 4°C. The cell lysis samples were mixed with reaction buffer and incubated with the substrate at 37°C for 2 h. A fluorescence microplate reader was used to measure the absorbance of the solution at excitation and emission wavelengths of 328 and 460 nm, respectively.

### Nuclear and Cytoplasmic Protein Extraction and Western Blot Assay

For total protein extraction, the GES-1 cells were treated as described in “Cells and treatment” section, washed twice with cold PBS, and lyzed with precooled RIPA (1% protease inhibitor) on ice for 15 min. The cells were scraped, collected into vials, and placed on ice for another 15 min for cell lysis. Next, the samples were sonicated at 300 W for 10 s at 5 s intervals for 2 min. The samples were centrifuged at 10,000× *g* for 10 min at 4°C, and the supernatant was collected for BCA assay. For nuclear and cytoplasmic protein extraction, the cell protein samples were extracted following the instructions of the NE-PER™ Nuclear and Cytoplasmic Extraction Reagents kit (Thermo Fisher, United States). Exactly 30 μg (4 μg for nuclear and cytoplasmic protein) of the protein samples was loaded onto 4–20% SurePAGE™ gels (Genscript, China) for electrophoresis and then transferred onto PVDF membranes using eBlot L1 *Trans*-Blot (Genscript, China). The membranes were blocked with 5% nonfat-dried milk and then incubated with anti-LC3 (1:1,000, CST, 12741S, United States), anti-P62 (1:1,000, CST, 39749S, United States), anti-TFEB (1:2000, Bethyl Laboratories, A303–673A, United States), and anti-β-actin (1:5,000, SAB, 21,338, United States) antibodies overnight at 4°C. The following day, the membranes were incubated with the secondary antibodies (CST, 7074S for anti-rabbit, 7076S for anti-mouse, United States) for 2 h at room temperature. Finally, the blots were treated with ECL reagent (Millipore, United States), observed under a chemiluminescence apparatus (Bio-rad, United States), and analyzed using ImageJ software.

### miRNA Chip Assay

GES-1 cells were cultured in a six-well plate at a density of 1.5×10^6^ cells/well. The cells were allowed to adhere to the walls of the wells for 12 h and then treated with DMSO (control) or 50 μM PA for 12 h. The cells were subsequently collected with Trizol (Life Technology, United States), and miRNA chip assay was performed by RIBOBIO Co.

### Nuclear and Cytoplasmic RNA Extraction and Quantitative Real-Time PCR Assay

GES-1 cells were infected with *H. pylori* (MOI = 100:1) for 6, 12, or 24 h or treated as described in “Cells and treatment” section. Total RNA samples were extracted using the RNApure Tissue and Cell Kit (CWBIO, China), while miRNA samples were extracted using the miRNA Purification Kit (CWBIO). Nuclear and cytoplasmic RNA samples were isolated using the Cytoplasmic and Nuclear RNA Purification Kit (Norgen, Canada). The purity of the nuclear RNA was determined using U2 snRNA (as a nuclear marker) and S14 (as a cytoplasmic marker).

The quality of the RNA samples was determined using a Nanodrop 2000 instrument (Thermo Scientific, United States), and samples with A_260_/A_280_ between 1.9 and 2.1 were considered to be of good quality. cDNA was generated using 1 μg of RNA with the FastKing-RT SuperMix Kit (TIANGEN, China), and real-time fluorescence quantitative qPCR assay was subsequently performed using Talent qPCR PreMix (TIANGEN, China). Here, GADPH was selected as the internal reference. The miRcute Plus miRNA First-Strand cDNA Kit (TIANGEN, China) was used to produce cDNA from the miRNA samples via the tailing reaction method, and the miRcute Plus miRNA qPCR Kit (TIANGEN, China) was subsequently used to explore the expression of the miRNA. U6 was selected as the internal reference for the miRNA assay (Supplementary Material S4).

### Statistical Analysis

Results are expressed as mean ± SD. Enumeration data were analyzed by one-way ANOVA followed by Dunnett’s test or the LSD test to compare differences among groups. The chi-squared test was used to analyze enumerated data. *p* < 0.05 was considered to indicate statistical significance.

## Results

### *H. pylori* May Achieve Long-Term Infection Through Intracellular Invasion

**FIGURE 2:**
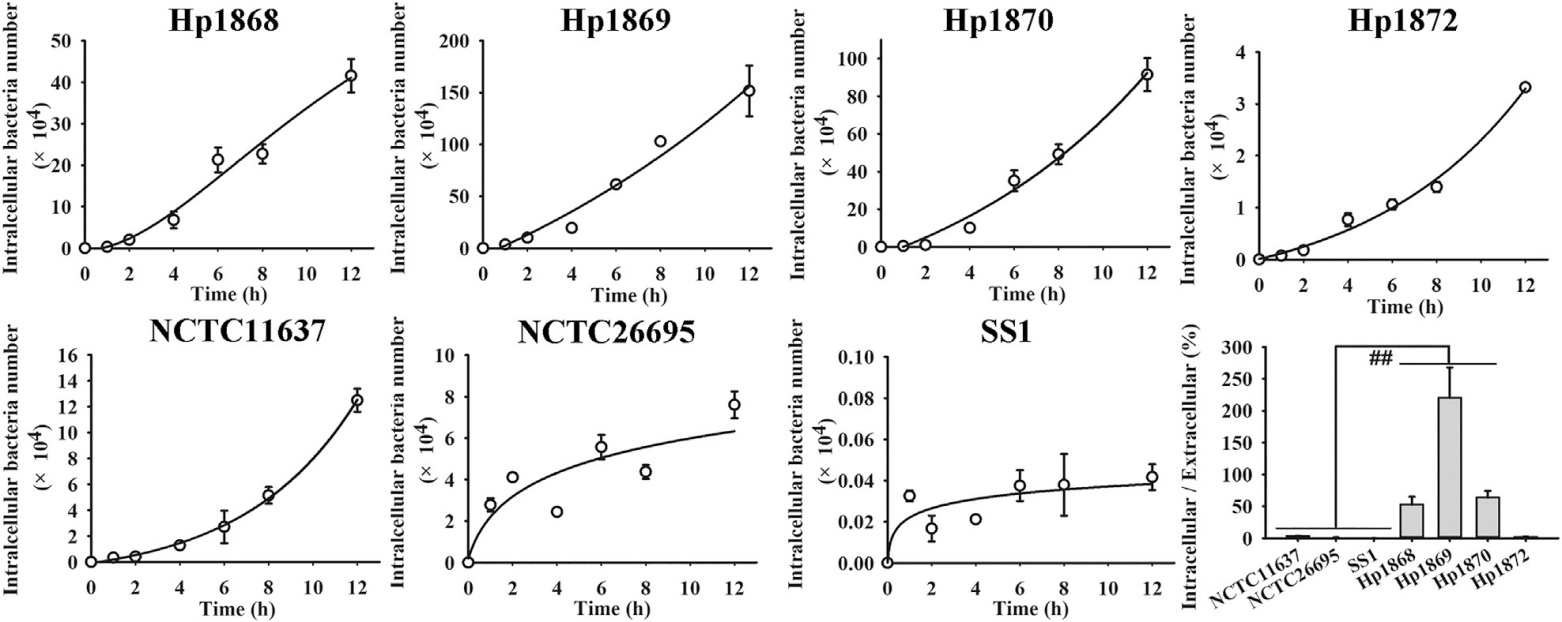
Time-dependent invasion of different *H. pylori* strains. The invasion rates of standard and clinical *H. pylori* strains increased over 12 h. The invasion rates of Hp 1868 (52.93 ± 24.23%), Hp 1869 (220.31 ± 95.68%), and Hp 1870 (63.88 ± 20.77%) were significantly higher than those of the standard strains NCTC11637 (3.46 ± 0.35%), NCTC26695 (1.24 ± 0.49%), and SS1 (0.01 ± 0.002%). **p < 0.01, *n* = 6.

The time-dependent invasion of different *H. pylori* strains was studied on the basis of the co-culture and gentamycin protection assays (Figure 2; Supplementary Material S5). The invasion of all strains continually increased over 12 h of culture. The intracellular/extracellular bacterial ratios of the three clinical strains, (i.e. Hp 1868, Hp 1869, and Hp 1870) were significantly higher than those of the three standard strains, (i.e. NCTC11637, NCTC26695, and SS1; *p* < 0.01). The intracellular/extracellular bacterial ratios obtained indicate that some clinical strains show stronger cell invasive ability than standard strains. Thus, intracellular invasion may be a strategy for the long-term infection of *H. pylori*.

### PA Ameliorates the Effects of *H. pylori* Infection and the Intracellular *H. pylori* Burden on GES-1 and MKN45 Cells

**FIGURE 3:**
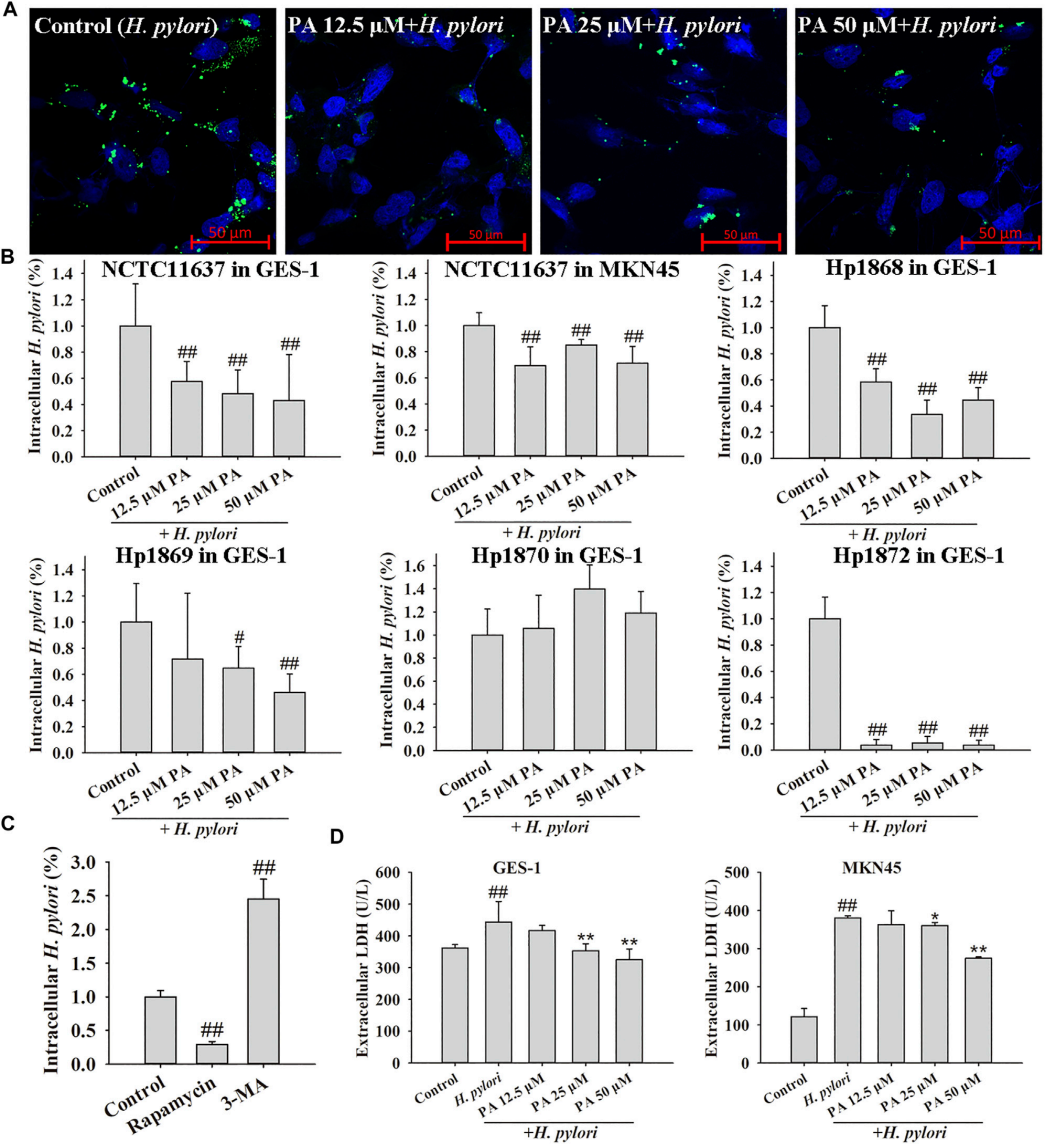
Effects of PA, an autophagy agonist (rapamycin), and an autophagy inhibitor (3-MA) on intracellular *H. pylori* survival. **(A)** Representative confocal images of intracellular *H. pylori* treated with PA. **(B)**. Effects of PA on the intracellular *H. pylori* strains NCTC11637, Hp 1868, Hp 1869, Hp 1870, and Hp 1872. The control group includes cells infected with *H. pylori* without treatment (*n* = 6). **(C)** Rapamycin reduced the number of intracellular *H. pylori* NCTC11637 whereas 3-MA increased the intracellular number of *H. pylori* NCTC11637 in GES-1 cells. The control group includes cells infected with *H. pylori* without treatment (*n* = 3). **(D)** LDH release from GES-1 and MKN45 cells increased following *H. pylori* infection. PA (25 and 50 μM) protected these cells from *H. pylori* infection (*n* = 6). #*p* < 0.05, ##*p* < 0.01, compared with the control group. *p < 0.05, **p < 0.01, compared with the *H. pylori* group.

In the intracellular *H. pylori* survival assay (Figure 3B), PA treatment (12.5, 25, or 50 μM) significantly reduced the numbers of *H. pylori* NCTC11637, Hp 1868, and Hp1872 in GES-1 cells and the number of *H. pylori* NCTC11637 in MKN45 cells. Treatment with 25 or 50 μM PA also decreased the number of *H. pylori* Hp1869 in GES-1 cells (*p* < 0.01, *p* < 0.05). However, PA showed no obvious influence on the invasion of Hp 1870. Visual and similar tendency (NCTC11637 in GES-1 cells) was observed by confocal microscopy, as shown in Figure 3A. Treatment with the autophagy agonist rapamycin reduced the number of intracellular *H. pylori*, whereas treatment with the autophagy inhibitor 3-MA increased the number of intracellular bacteria found (*p* < 0.01). High levels of extracellular LDH reflect cell damage, and results indicated that treatment with 25 or 50 μM PA could protect GES-1 cells from *H. pylori* infection (*p* < 0.01). In addition, treatment with 50 μM PA reduced the level of extracellular LDH in *H. pylori*-infected MKN45 cells (*p* < 0.01, *p* < 0.05).

### PA Increases Xenophagy After *H. pylori* Infection

**FIGURE 4:**
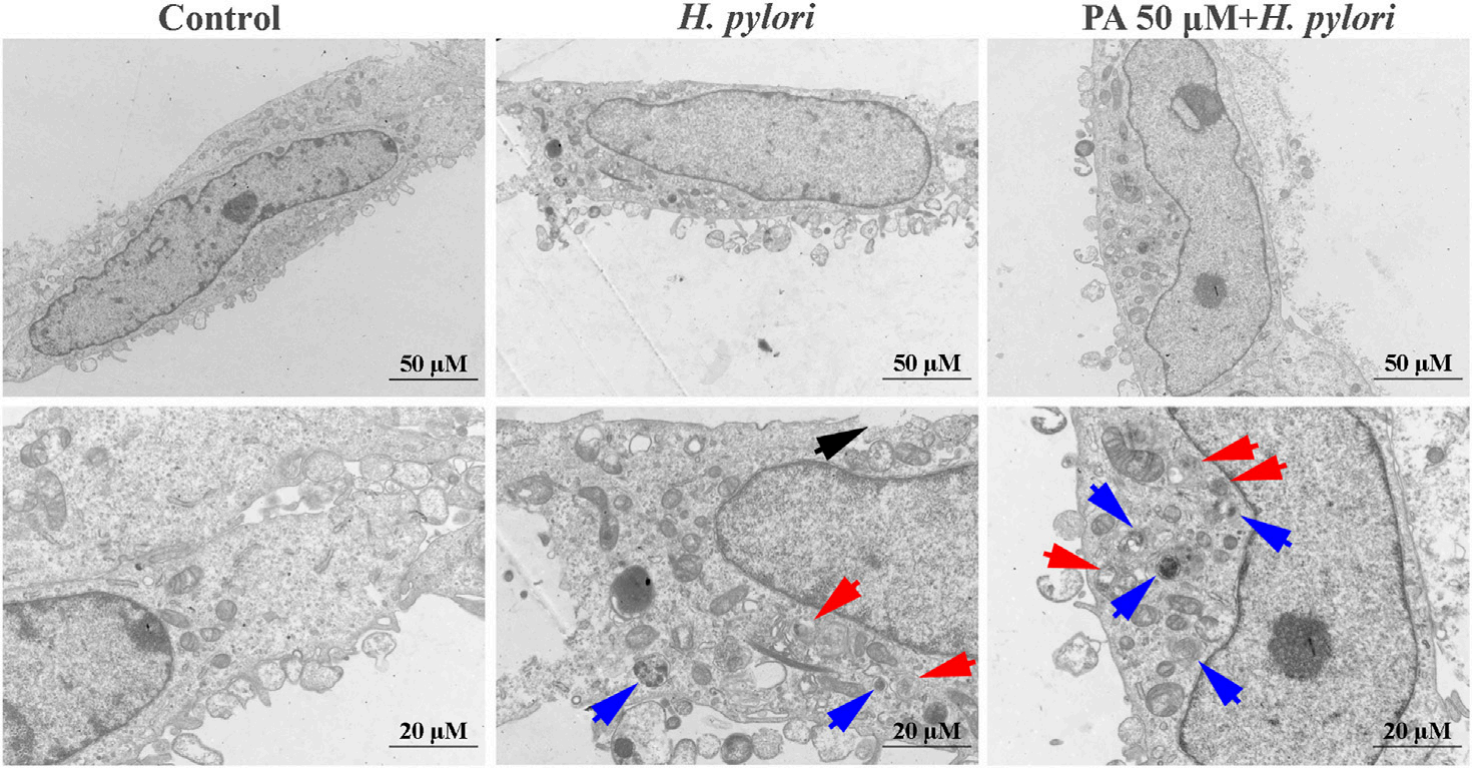
Representative TEM micrographs of the ultrastructures of *H. pylori*-infected GES-1 cells. Double-membraned autophagosomes, red arrows; single-membraned autolysosomes, blue arrows; damaged cell membranes, black arrows.

As shown in Figure 4, obvious cell membrane damage could be observed following treatment with *H. pylori* (black arrows). Double-membraned autophagosomes (red arrows) and single-membraned autolysosomes (blue arrows) were also noted. Treatment with PA, especially at a dose of 50 μM, significantly increased autophagic flux levels, as observation of a mass of autophagosomes and autolysosomes.

### PA Promotes Xenophagy Against Intracellular *H. pylori*

**FIGURE 5:**
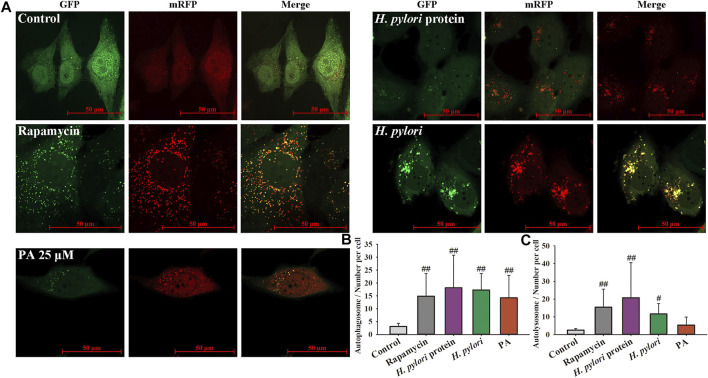
*H. pylori* infection induced xenophagy in GES-1 cells. mRFP-LC3-GFP adenovirus was adopted to label autophagosomes and autolysosomes. GFP fluorescence is quenched when autophagosomes fuse with lysosomes. Merged yellow dots indicate autophagosomes, and autolysosome numbers were calculated as number of red dots minus number of yellow dots. **(A)** Representative images of the xenophagy state after different treatments for 12 h. **(B)** Quantitative results of autophagosomes. **(C)** Quantitative results of autolysosomes. #*p* < 0.05, ##*p* < 0.01, compared with the control group, *n* = 4.

**FIGURE 6:**
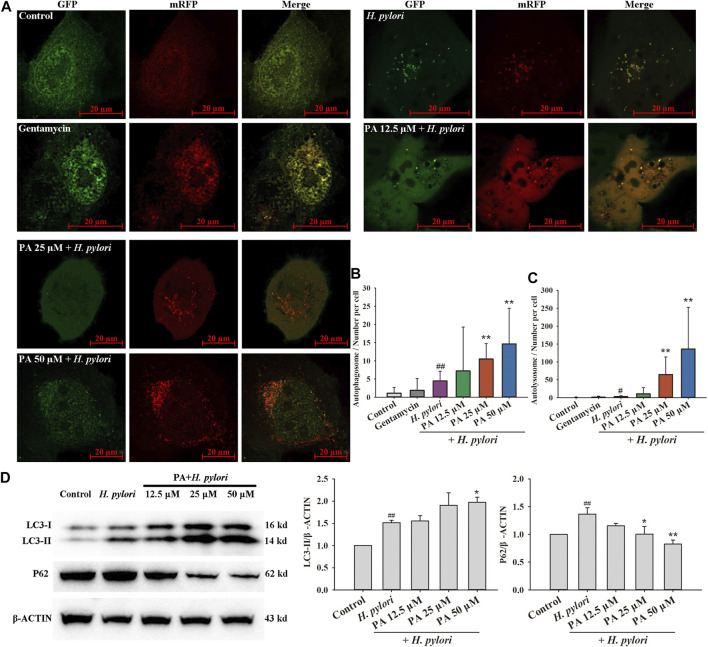
Effects of PA on *H. pylori*-induced xenophagy. **(A)** Representative images of xenophagy after treatment with gentamycin and *H. pylori* with or without PA. **(B)** Quantitative results of autophagosomes (*n* = 4). **(C)** Quantitative results of autolysosomes (*n* = 4). **(D)** Representative Western blot results of LC3 and P62. Upregulation of LC3-II confirmed the occurrence of xenophagy, while downregulation of P62 revealed the fusion of lysosomes with autophagosomes and digestion of the content (*n* = 5). The results indicate that PA improves the xenophagic flux and promotes digestion. ##*p* < 0.01, compared with the control group. *p < 0.05, **p < 0.01, compared with the *H. pylori* group.

As shown in Figure 5, the numbers of autophagosomes and autolysosomes significantly increased following treatment with rapamycin, *H. pylori* (MOI = 100:1), and *H. pylori* protein (6.25 μg/ml). Treatment with PA slightly increased the number of autophagosomes (*p* < 0.01, *p* < 0.05). The numbers of autophagosomes in the *H. pylori* and *H. pylori* protein groups were similar, but the number of autolysosomes in the *H. pylori* protein group was nearly twice that in the *H. pylori* group. This result indicates that intact *H. pylori*, but not the protein only, may cause the severe destruction of the autophagic flux. As shown in Figure 6A, gentamycin treatment had no effect on autophagy, and extracellular bacterial clearance clearly downregulated the autophagy level induced by *H. pylori*. Treatment with 25 or 50 μM PA remarkably increased the numbers of autophagosomes and autolysosomes induced by *H. pylori* infection (Figure 6B,C; *p* < 0.01). Similar results were observed in the Western blot assays. As shown in Figure 6D, although *H. pylori* infection induced the overexpression of LC3-II and P62 (*p* < 0.01), LC3-II levels further increased following 50 μM PA treatment (*p* < 0.05). P62 levels decreased in the 25 and 50 μM PA groups (*p* < 0.01, *p* < 0.05).

### PA May Defend Against Intracellular *H. pylori* Infection by Downregulating miR-30 b and miR-30c

**FIGURE 7:**
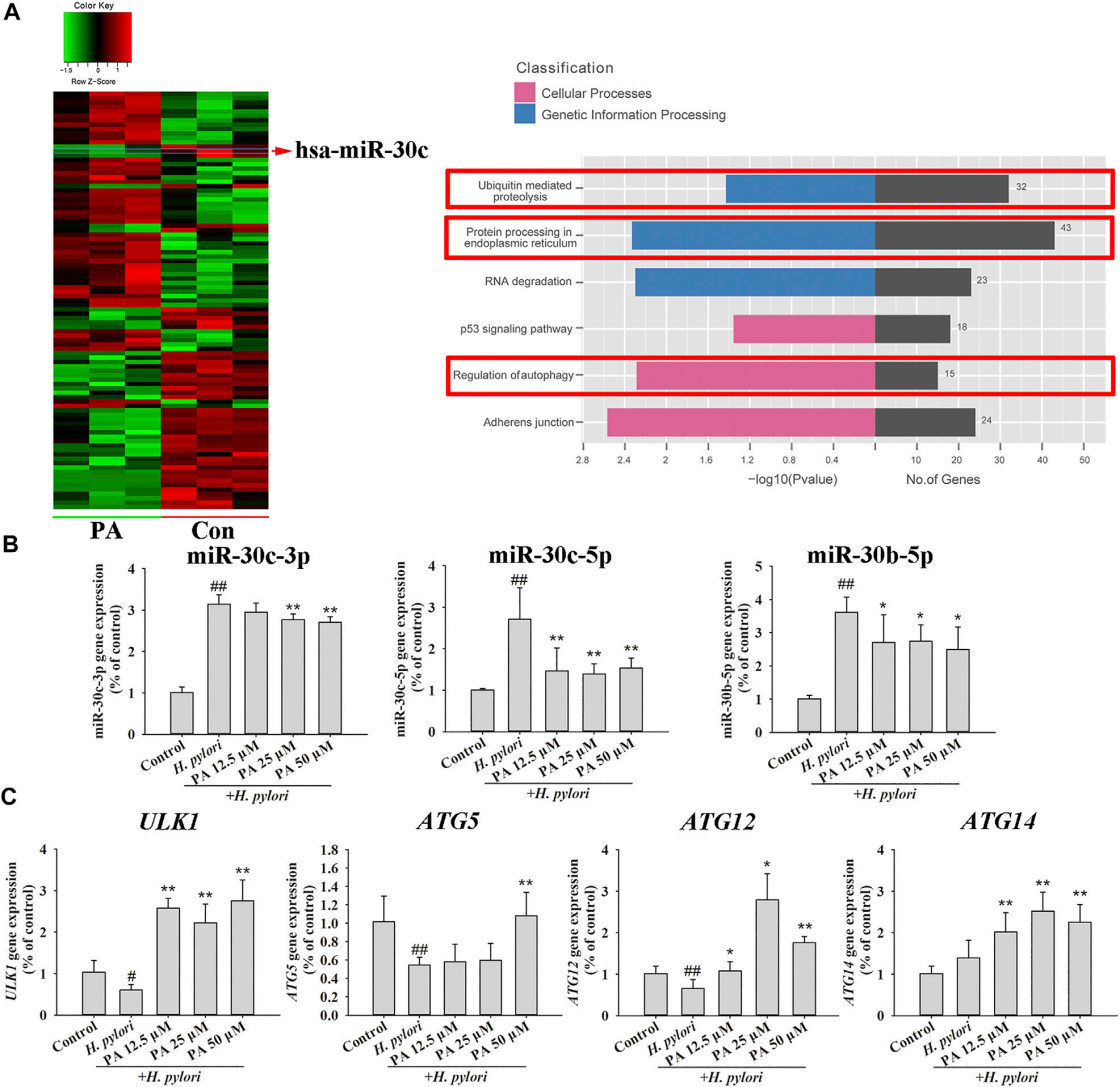
MiRNA regulation by PA promoted xenophagy. **(A)** MiRNA chip assay was performed on PA-treated or untreated GES-1 cells, and KEGG analysis was conducted to assess differentially expressed miRNAs (*n* = 3). **(B)** Quantitative gene expression results of miR-30c-3p, miR-30c-5p, and miR-30b-5p. **(C)** Quantitative gene expression results of *ULK1*, *ATG5*, *ATG12*, and *ATG14*. ##*p* < 0.01, compared with the control group. *p < 0.05, **p < 0.01, compared with the *H. pylori* group, *n* = 6.

The effect of PA on the miRNA expression of GES-1 cells was investigated by miRNA chip assay, and the results of KEGG analysis based on the differentially expressed miRNAs obtained revealed that PA may regulate several cellular processes, including ubiquitin-mediated proteolysis, protein processing in the endoplasmic reticulum, and autophagy (Figure 7A; Supplementary Material S6). The effects of PA on miR-30c-3p, miR-30c-5p, and miR-30b-5p were then investigated. The expression of miR-30c-3p, miR-30c-5p, and miR-30b-5p was remarkably upregulated by *H. pylori* infection (*p* < 0.01), but treatment with PA (12.5, 25, or 50 μM) significantly downregulated the expression of miR-30c-5p and miR-30b-5p (*p* < 0.01, *p* < 0.05). Moreover, treatment with 25 or 50 μM PA downregulated the expression of miR-30c-3p (Figure 7B; *p* < 0.01). The expression of the target genes of miR-30c-3p (ULK1 and ATG14), miR-30c-5p (ATG12), and miR-30b-5p (ATG5 and ATG12) were further analyzed (Figure 7C). The results revealed that *H. pylori* infection decreases the gene expression of *ULK1*, ATG5, and ATG12 (*p* < 0.01, *p* < 0.05), but treatment with 12.5, 25, or 50 μM PA increases the gene expression of ULK1, ATG12, and ATG14 (*p* < 0.01, *p* < 0.05). Moreover, treatment with 50 μM PA upregulated the gene expression of *ATG5* (*p* < 0.01).

### PA Increases Lysosome Activity and Promotes Intracellular *H. pylori* Digestion by Downregulating miR-30b-5p in the Nucleus

**FIGURE 8:**
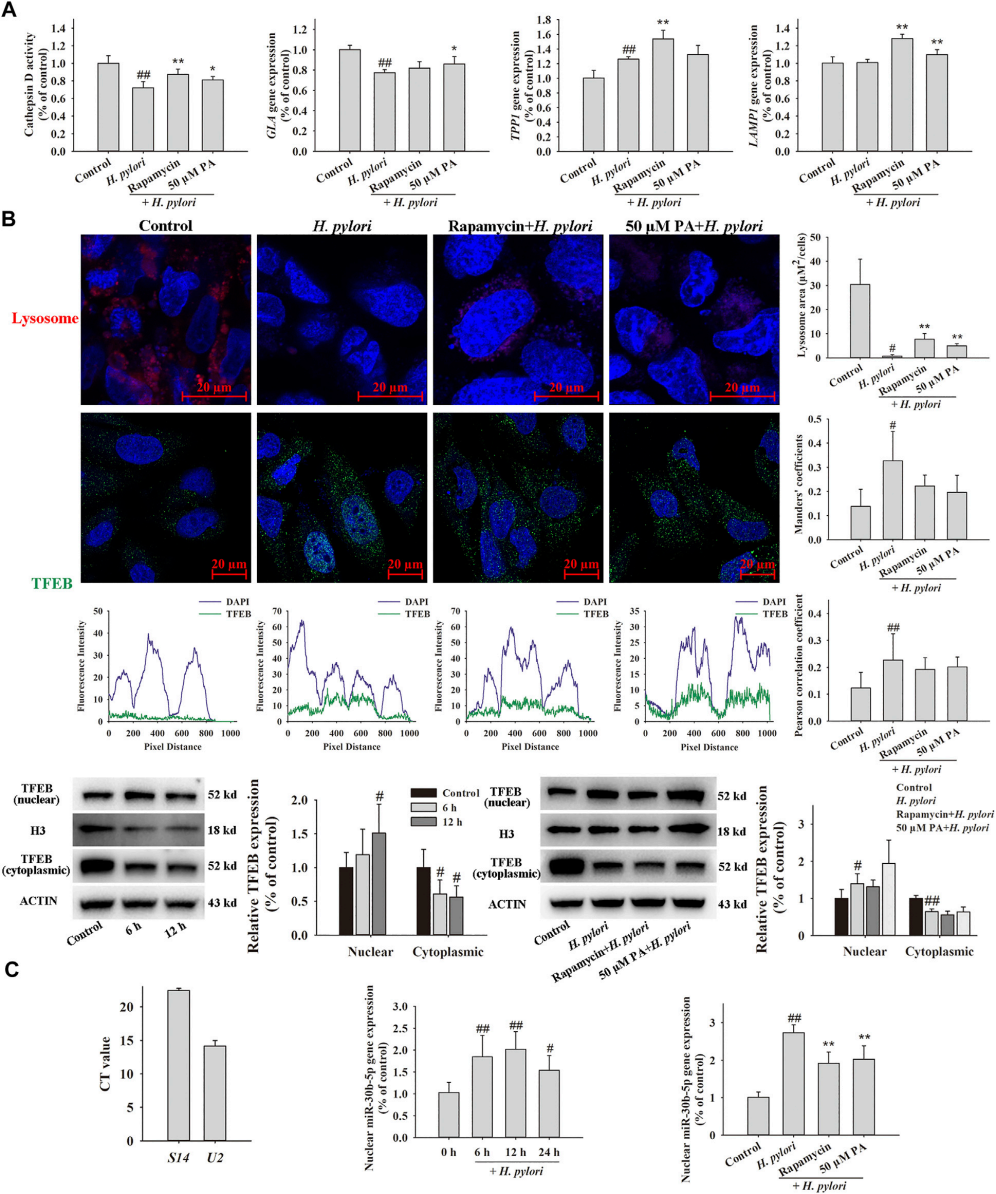
Effects of PA on lysosome function. GES-1 cells were pretreated with rapamycin or PA for 12 h, infected with *H. pylori* for 12 h (except for the time-dependent assay), and then analyzed by qRT-PCR, Western blot, and immunofluorescence assays. **(A)** Effects of PA on *H. pylori*-induced lysosome activity, including cathepsin D activity (*n* = 8), representative lysosome gene expression, and representative images of LysoTracker Red DND-99-labeled lysosome (*n* = 5). **(B)** Effects of PA on the lysosome mediator TFEB, including representative images of TFEB colocalized with nuclear DAPI and the corresponding quantitative data (*n* = 8), representative Western blots (*n* = 5), and quantitative data of nuclear and cytoplasmic TFEB. **(C)** Effects of PA on the nuclear transfection of miR-30b-5p, including confirmation of the successful isolation of nuclear RNA, time-dependent changes in nuclear miR-30b-5p expression, and downregulation of nuclear miR-30b-5p expression following PA treatment (*n* = 6).

Assays of lysosome activity (Figure 8A) indicated that *H. pylori* infection reduces the activity of cathepsin D (*p* < 0.01). Downregulation of *galactosidase alpha* (GLA) gene expression (*p* < 0.01) and upregulation of *tripeptidyl peptidase one* gene expression (*p* < 0.01) were observed after *H. pylori* infection; these genes are representative genes of lysosomes. Treatment with 50 μM PA significantly increased the expression of *GLA* and lysosome-associated membrane protein-1 (*p* < 0.01, *p* < 0.05). Acidic lysosomes were identified using LysoTracker Red DND-99, and the results indicated that *H. pylori* infection nearly completely eliminates the acidic lysosomes of GES-1 cells. Pretreatment with rapamycin and 50 μM PA partly recovered these lysosomes, and differences between these and the *H. pylori* infection groups were statistically significant (*p* < 0.01). In the co-localization assay (Figure 8B), the nuclear translocation of TFEB obviously increased in *H. pylori*-infected GES-1 cells, as reflected by alterations in the Mander and Pearson correlation coefficients. However, no changes in the location of TFEB were noted after treatment with rapamycin and *p*A. The time-dependent transfection of TFEB to the cell nucleus was assessed by Western blot assay (*p* < 0.01, *p* < 0.05), which revealed no distinct trend of nuclear TFEB translocation. The isolated nuclear RNA was purity, because the difference in CT value exceeded 5, as shown in Figure 8C. The transfection of miR-30b-5p to the cell nucleus remarkably increased 6, 12, and 24 h after *H. pylori* infection (*p* < 0.01, *p* < 0.05). Rapamycin and PA significantly reduced the amount of endonuclear miR-30b-5p (*p* < 0.01).

## Discussion

The increased resistance of *H. pylori* to antibiotics affects the clearance rate of triple and quadruple therapies worldwide, especially in developing countries such as China. Indeed, the resistance rate of the pathogen to metronidazole, clarithromycin, and levofloxacin has been reported to be as high as 78.2, 22.1, and 19.2%, respectively (Liu et al., 2018). The invasion of *H. pylori* into gastric epithelial cells exponentially increases the tolerance of the bacteria against drugs, and low-dose or inappropriate antibiotic treatment could produce an unfavorable external environment that may further promote *H. pylori* invasion. Therefore, sufficient first-time treatment is crucial. The rates of secondary resistance of *H. pylori* to multiple antibiotics may attributed to the high invasion phenomenon (Lyu et al., 2020). Our results of the comparative study indicated that some clinical strains have stronger invasive ability than standard strains, and the intracellular invasion may be a survival strategy of *H. pylori* because intracellular environments allow bacteria to avoid immune cells or exposure to antimicrobials (Thakur et al., 2019). Another research revealed that the invasive ratio of NCTC26695 is 2.5%, which is consistent with our results (Kwok et al., 2002). To date, whether the intracellular survival of *H. pylori* plays a role in pathopoiesia is unknown. And our lab has begun a clinical experiment to explore the influence of increasing invasion of *H. pylori* on patient illness and clinical therapy. Recent studies have revealed that impairment of the lysosomal calcium channel MCOLN1/TRPML1 by VacA can improve the intracellular survival ability of *H. pylori* (Capurro et al., 2020). In short, these findings strongly suggest that eradication of intracellular *H. pylori* should receive attention during clinical treatment.

Intracellular survival contributes to the drug tolerance of *H. pylori* (Lai et al., 2006), and the activation of xenophagy by *H. pylori* produce autophagosome, which provide a niche for the intracellular survival of *H. pylori*, thus increasing the frequency of drug-resistant bacteria (Greenfield and Jones, 2013). The drug-resistant *H. pylori* accompanied with strong invasive ability would bring a lot of trouble to clinical therapy undoubtedly. Besides, unfavorable environments with low concentrations of antibiotics may promote bacterial invasion. Pretreatment with PA decreased the intracellular number of *H. pylori*, reduced the release of LDH, and protected GES-1 cells. These findings demonstrate the promising potential clinical applications of PA in intracellular bacteria therapy. Immunofluorescence and gentamycin protection assays were adopted to analyze the intracellular number of *H. pylori*. Immunofluorescence images enable the detailed observation of intracellular bacteria. However, the time-consuming gentamycin protection assay provides more accurate data because dead bacteria can also be stained.

TEM revealed that *H. pylori* treatment could damage the cell membrane and increase the autophagic flux, whereas PA treatment could further improve it. Similar results were obtained in the adenovirus transfection assay, which showed that PA increases the abundance of autolysosomes and promotes the digestion of *H. pylori*. Previous studies revealed that the VacA secreted by *H. pylori* could react with low-density lipoprotein receptor-related protein 1 (LRP1) receptors in epithelial cells, which activates xenophagy (Chauhan et al., 2019). The bacteria then hijack the xenophagy process, inhibit autophagosome formation, and reduce cathepsin D in lysosomes, all of which contribute to the intracellular survival of *H. pylori* (Sit et al., 2020). *H. pylori* protein is sufficient for xenophagy activation (Zhu et al., 2017), but the invasion of intact *H. pylori* bacteria seems to lead to more lethal effects than exposure to the protein only.

MiRNA-30 families play a key role in *H. pylori*-related xenophagy. For example, the miRNAs miR-30b and miR-30 days have been directly associated with *H. pylori*-induced xenophagy (Tang et al., 2012; Zhang et al., 2019a). KEGG analysis of cellular processes and genetic information processing was performed on the basis of the results of miRNA chip assay, and findings indicated that PA affects ubiquitin, the endoplasmic reticulum, and autophagy. Further verification assay confirmed the effects of PA on miR-30c-3p, miR-30c-5p, and miR-30b-5p, and the related target genes. The time difference of gene and protein expression contribute to the confliction of downregulation of xenophagy gene (*ULK1*, *ATG5* and *ATG12*) and increment of xenophagy protein expression (LC3) (Schwanhäusser et al., 2011). MiR-30b-5p was proven to be a nuclear miRNA that can transfer into the nucleus and inhibit the function of TFEB (Guo et al., 2020). TFEB is the master regulator of autophagy and lysosomal biogenesis and transfers to the nucleus to modulate the autophagic flux (Yang et al., 2020). Our data suggest that the nuclear translocation of TFEB occurs during *H. pylori* infection, which contributes to the activation of xenophagy. However, the role of TFEB in lysosome modulation could be partly blocked by the upregulation of nuclear miR-30b-5p induced by *H. pylori*, which is one of the strategies for its intracellular survival. Rapamycin activates lysosome function by TRP channel, but not TFEB, which is consistent with our result (Zhang et al., 2019b). Collectively, the elimination of lysosome activity by *H. pylori* could be attributed to two factors. On the one hand, *H. pylori* urease converts urea into ammonium ions, which raises the pH of the intracellular environment and impairs lysosome function (Schwartz and Allen, 2006). On the other hand, the upregulation of nuclear miR-30b-5p blocks the activity of TFEB and inhibits lysosome function. Although no effect of PA on TFEB translocation was shown, the downregulation of miR-30b-5p may contribute to the improvement of lysosome function in the studied system.

PA, from *P. cablin* (Blanco) Benth (Labiatae), is a naturally occurring tricyclic sesquiterpene characterized with high absorptivity and membrane permeability. The compound also shows high cellular uptake and transport efficiency and has been demonstrated to exert therapeutic effects against *H. pylori*-induced gastritis (Lian et al., 2018). The high cell-membrane permeability of PA renders it as an effective agent against intracellular *H. pylori*. Our data revealed the bactericidal effects of PA on intracellular *H. pylori* via the ability of improving miR-30b/c-mediated xenophagy and TFEB-related lysosome function. This study provides an extensive assessment of the multiple therapeutic effects of PA, which may present synergistic effects in combination with other antibiotics against *H. pylori*, especially highly invasive intracellular strains. The results reflect the potential use of PA as a complementary therapy against this highly pathogenic bacterium.

## Conclusion

**FIGURE 9:**
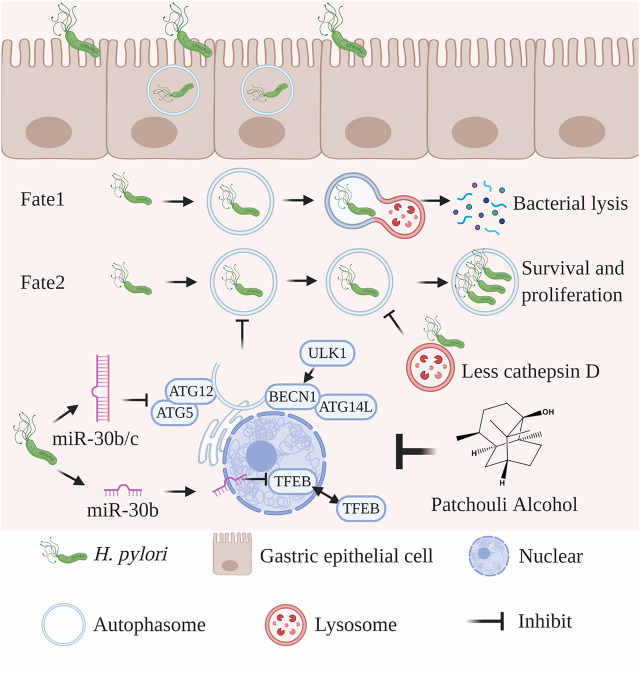
Proposed mechanism of PA against intracellular *H. pylori*. Some *H. pylori* bacteria could invade gastric epithelial cells. In the early stages of infection, *H. pylori* is enveloped by autophagosomes, which then fuse with lysosomes to form autolysosomes and digest the bacteria. As infection progresses, intracellular *H. pylori* secretes several toxic factors, such as VacA, to inhibit the xenophagic flux. The expression of miR-30c-3p, miR-30c-5p, and miR-30b-5p is typically upregulated by *H. pylori* infection and results in the downregulation of *ULK1*, *ATG5*, *ATG12*, and *ATG14* and inhibition of the formation of autophagosomes. MiR-30c-5p could be transferred to the nucleus to inhibit the functions of TFEB and lysosomes. PA upregulates the expression of miR-30c-3p, miR-30c-5p, and miR-30b-5p to promote the xenophagic flux and lysosome function. The figure was created using BioRender.com supported by the Postdoc plan.

The strong cell invasive ability is observed in some clinical *H. pylori* strain, and the eradication of strongly invasive *H. pylori* should receive attention during clinical treatment. The relationship between clinical outcome and the cell invasive of *H. pylori* is still indistinct, which should be further illustrated based on clinical research. Moreover, PA can act against invasive *H. pylori* based on the improvement of miR-30b/c mediated xenophagy (Figure 9).

## Data Availability

The raw data supporting the conclusions of this article will be made available by the authors, without undue reservation, to any qualified researcher.
