# Supplementary material for: Clinical Strains of Helicobacter pylori With Strong Cell Invasiveness and the Protective Effect of Patchouli Alcohol by Improving miR-30b/C Mediated Xenophagy
Source: Front Pharmacol. 2021 Apr 30;12:666903. doi: 10.3389/fphar.2021.666903 (PMC8120110; doi:10.3389/fphar.2021.666903)
Supplement: Supplementary file 1 [file DataSheet1.PDF]

Supplementary Material 1

GES-1 and MKN45 cells were cultured in 6-well plate, and co-cultured with *H. pylori* NCTC11637 strain for 2, 4 and 6 h, respectively. The release of LDH in different time was measured using the kit from Jiancheng Company, Nanjing, China. And our results showed that the LDH release were increased after 4 h, indicating the cell damage was occurred after 4 h co-culture. As shown in Supplementary Fig.1.

A

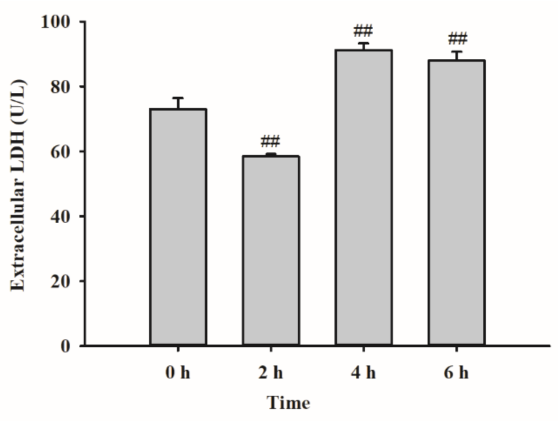

B

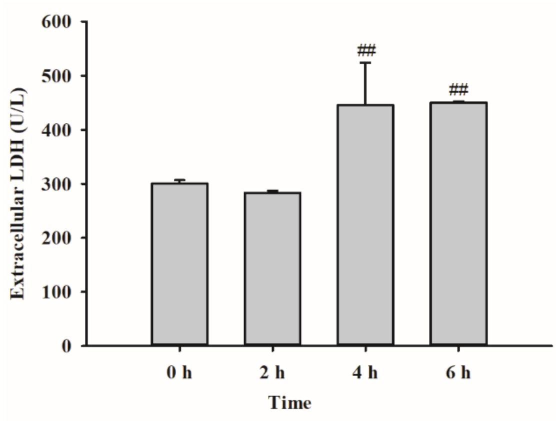

Fig. S1 (A: *H. pylori* NCTC11637 infected GES-1; B: *H. pylori* NCTC11637 infected MKN45; n=3)

Supplementary Material 2

Different concentrations of mRFP-LC3-GFP adenovirus were used to infected GES-1 cells, and the results indicated that 6.3×10^{7} PFU/ml mRFP-LC3-GFP adenovirus was high-efficiency for GES-1 transfection without obvious damage in 24 h. The transfection rate reach to 80% in those assays, meeting the experiment requirement.

A **B**

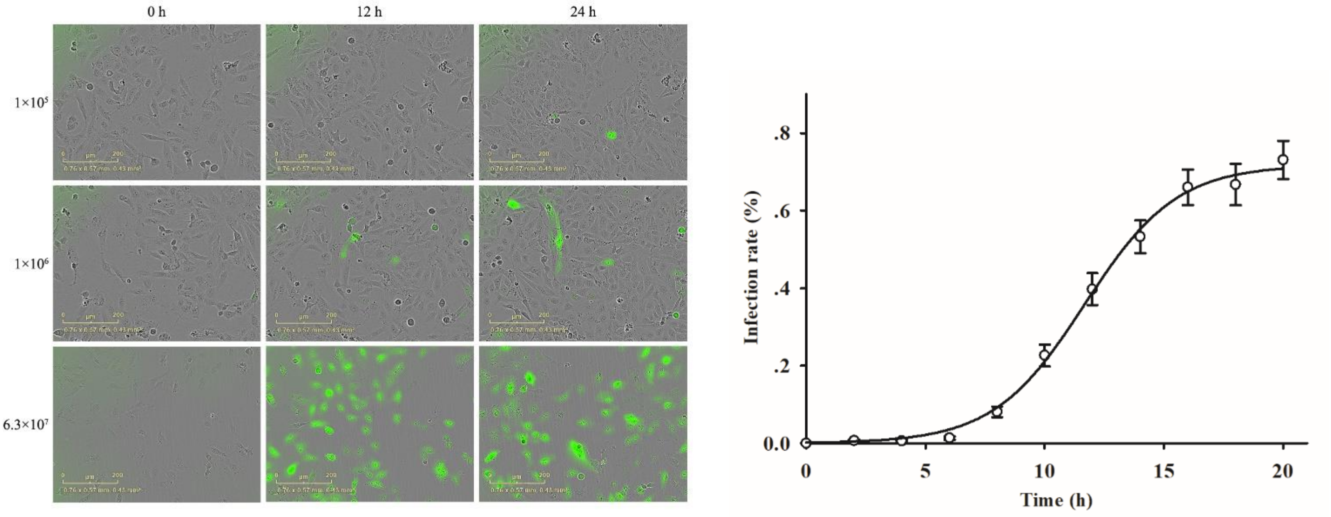

Fig. S2 (A: Time- and concentration- dependent transfection rate; B: The transfection rate recorded by IncuCyte ZOOM Real-Time Live-Cell Imaging System, Essen Bioscience; n=6)

Supplementary Material 3

The GES-1 cell proliferation was recorded by IncuCyte ZOOM Real-Time Live-Cell Imaging System (Essen Bioscience). In supplementary Fig.3 A, the influences of *H. pylori* (MOI=100:1) or *H. pylori* protein (6.25 μg/ml) on GES-1 cell proliferation were observed. And the results showed that *H. pylori* and *H. pylori* protein inhibited GES-1 cell proliferation after 24 and 12 h, respectively. The sudden increment of the result in *H. pylori* infection group was considered to be the additional of *H. pylori*, since the proliferation of cell was calculated by area recorded by the equipment. Different concentrations of PA (12.5, 25 and 50 μM) haven't showed any impact on GES-1 cell proliferation.

A

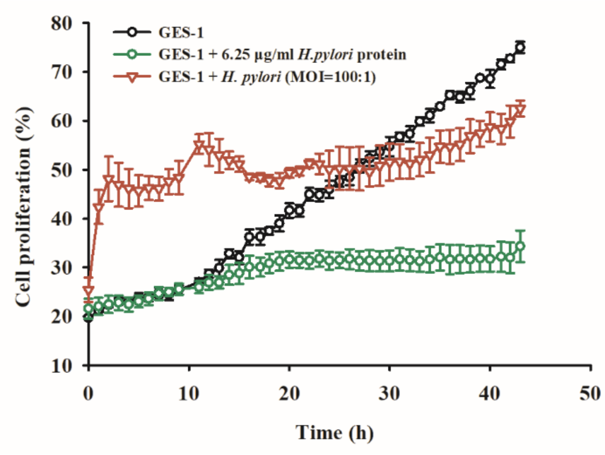

B

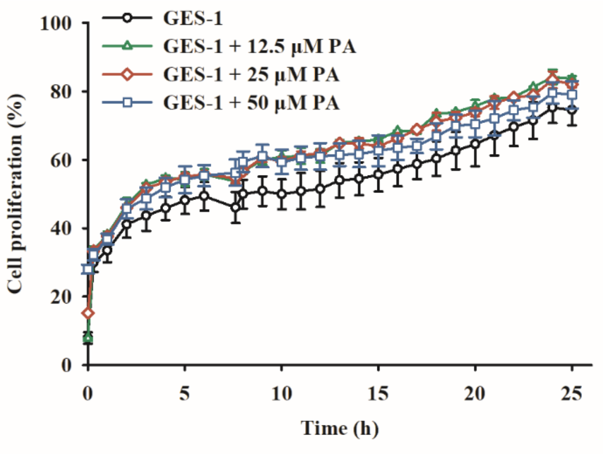

Fig. S3 (A: Cell proliferation with *H. pylori* (MOI=100:1) or *H. pylori* protein (6.25 μg/ml) treatment; B: Cell proliferation with PA treatment; n=6)

Supplementary Material 4

The primers used in this experiment were listed in Table S1. The primers for miRNA were purchased from TIANGEN company, and the catalog number, miRbase database number and miRNA sequence were shown in Table S2.

Table S1 The primers for Q-PCR assay

Gene	**Forward primer (5'-3')**	**Reverse primer (5'-3')**
ATG5	GCAGATGGACAGTTGCACACAC	GAGGTGTTTCCAACATTGGCTCA
ATG12	AGTAGAGCGAACACGAACCATCC	AAGGAGCAAAGGACTGATTCACATA
ATG14L	TGTACCTGGTCAGTCCAAGCTC	CAGGTCGGTTTCTTCATCGCTG
GADPH	CCACATCGCT CAGACACCAT	GGCAACAATATCC ACTTTACCAGAGT
GLA	AGCCAGATTCCTGCATCAGTG	ATAACCTGCATCCTTCCAGCC
LAMP1	ACGTTACAGCGTCCAGCTCAT	TCTTTGGAGCTCGCATTGG
S14	GGCAGACCGAGATGAATCCTC	CAGGTCCAGGGGTCTTGGTCC
TFEB	GACTCAGAAGCGAGAGCTAACA	TGTGATTGTCTTTCTTCTGCCG
U2	TTGGGAATTCTCAAGTGTAGTATCTGTTCTTAT	AGGCGAATTCGCGATGCGCTCGCCTTCGCGCCC
***ULK1*(*ATG1*)**	GCAAGGACTCTTCCTGTGACAC	CCACTGCACATCAGGCTGTCTG

Table S2 Information for miRNA

Gene	**Catalog number from TIANGEN**	**miRbase number**	**miRNA sequence**
**hsa-miR-30c-5p**	CD201-0338	MIMAT0000244	UGUAAACAUCCUACACUCUCAGC
**hsa-miR-30c-3p**	CD201-0405	MIMAT0004674	CUGGGAGAGGGUUGUUUACUCC
**hsa-miR-30b-5p**	CD201-0337	MIMAT0000420	UGUAAACAUCCUACACUCAGCU
**hsa-miR-U6**	CD201-0145	One of snRNAs hasn't recorded in miRbase	

Supplementary Material 5

Firstly, the extracellular bacteria eradication efficiency of gentamycin was verified. In details, the *H. pylori* strains growth in BHI supplied with 10% FBS were treated with 100 μg/ml gentamycin for 6 h. And then, the medium was removed and cultured in Columbia agar base with 5% sheep blood at triple-gas incubator for 5 d to form colonies. The results showed that 100 μg/ml gentamycin could completely clear the extracellular bacteria at 6 h. As shown in Fig. S5-1.

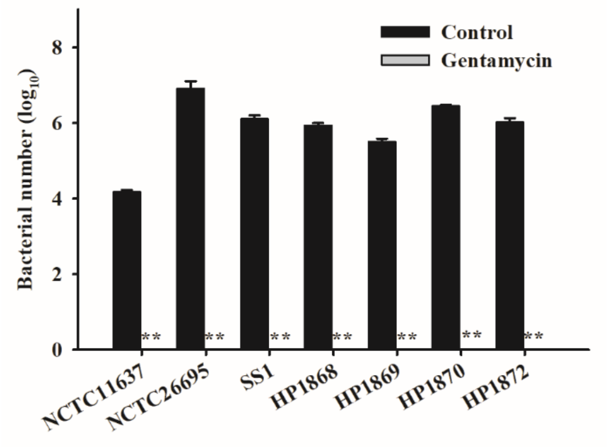

Fig. S5-1

The time-dependent invasion of *H. pylori* NCTC11637 was observed by confocal microscopy. The cell nucleus, GES-1 and *H. pylori* were stained with Hoechst33342, CMFDA and CMTPX, respectively. Briefly, *H. pylori* NCTC11637 were re-suspended in BHI containing 10% FBS, and the CMTPX (AAT Bioquest, 136832-63-8) was added to stain the bacteria (12.5 μM) in tri-gas incubator shaken at 120 rpm overnight. The GES-1 cells were re-suspended in HBSS and stained by 20 μM CMFDA (AAT Bioquest, 136832-63-8) and 0.1% Hoechst33342 in CO_2 incubator. 30 min later, the cells were cultured in glass-bottomed dish, and co-cultured with the pre-stained bacteria. After 1, 2, 4, 8 and 12 h, 100 μg/ml gentamycin was recruited to clear extracellular bacteria, and the cells were mount by Fluoromount-G^{TM} (Invitrogen, USA) until confocal observation. As shown in Fig. S5-2.

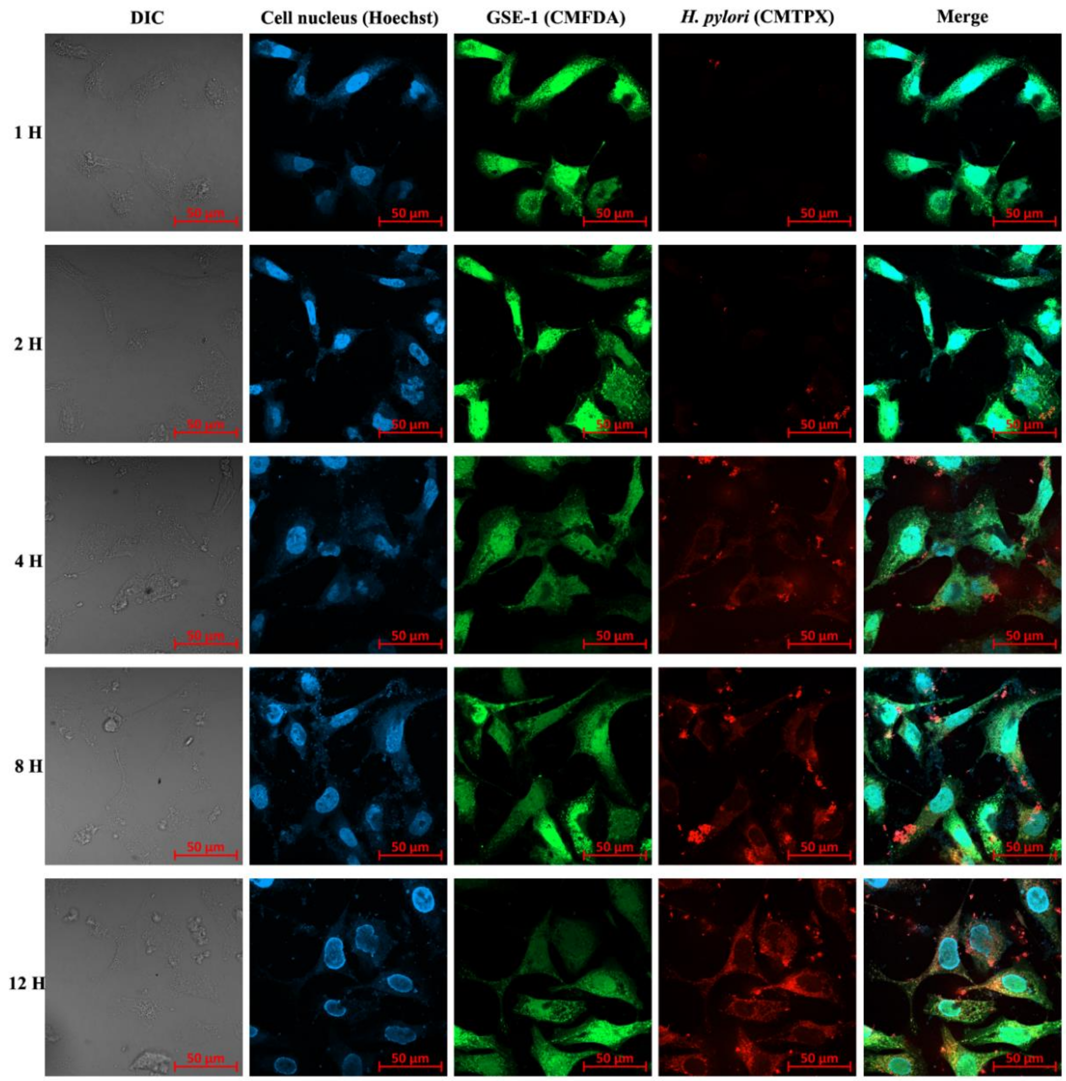

Fig. S5-2

The time-dependent invasive ratios of intracellular/extracellular bacteria were shown in Supplementary Fig. S5-3. Gentamycin protection assay was adopted to count the bacteria. The supernatants and cell lysis containing bacteria were collected at 1, 2, 4, 6, 8 and 12 h, and cultured in Columbia blood agar. The colonies formed 4 days later were recorded and analyzed.

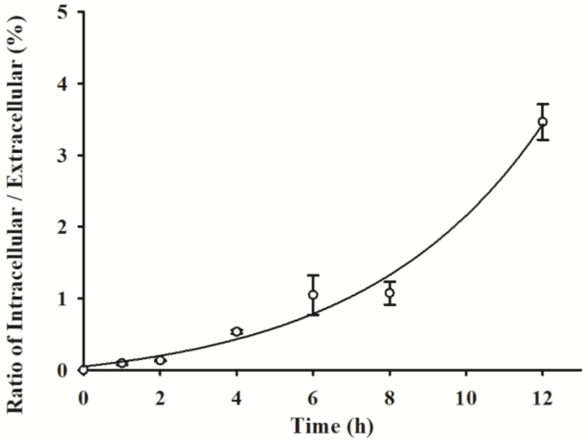

Fig. S5-3-1 NCTC11637

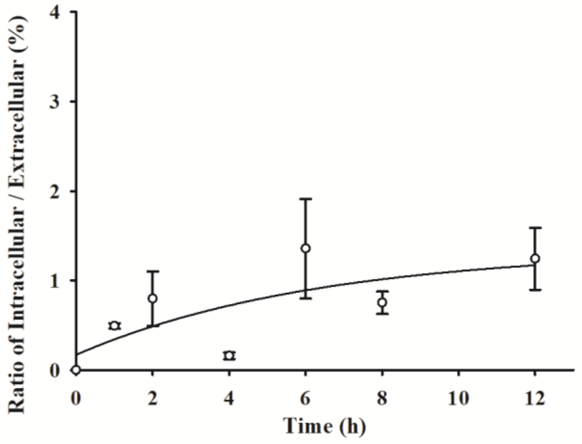

Fig. S5-3-2 NCTC26695

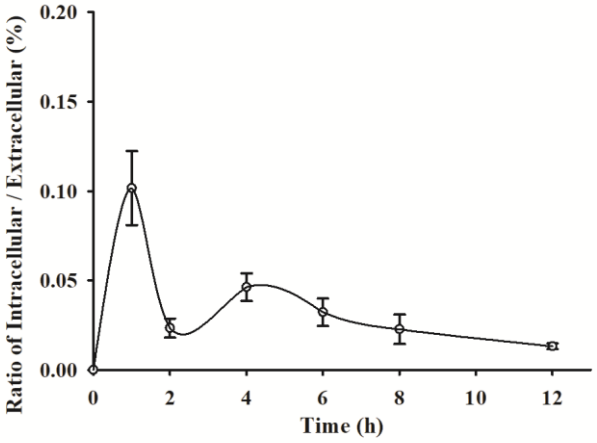

Fig. S5-3-3 SS1

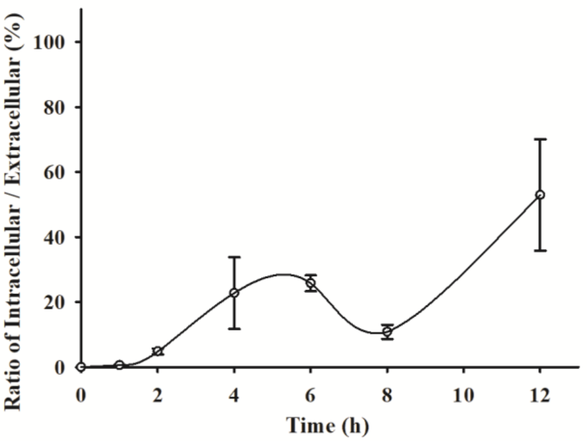

Fig. S5-3-5 Hp1868

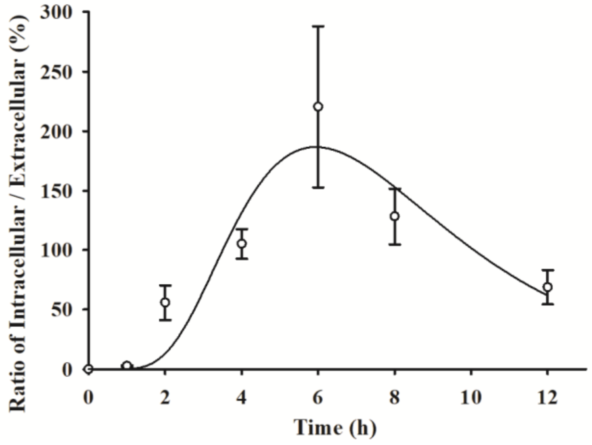

Fig. S5-3-6 Hp1869

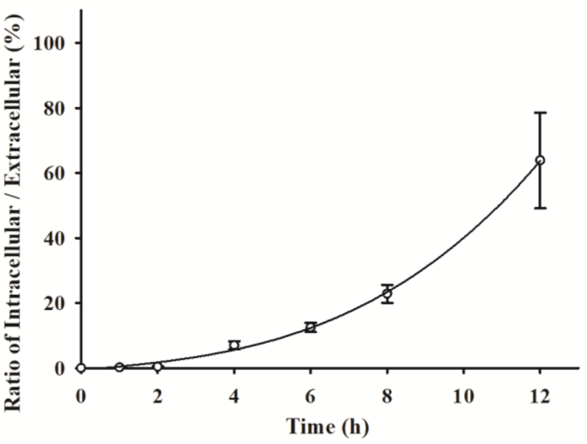

Fig. S5-3-7 Hp1870

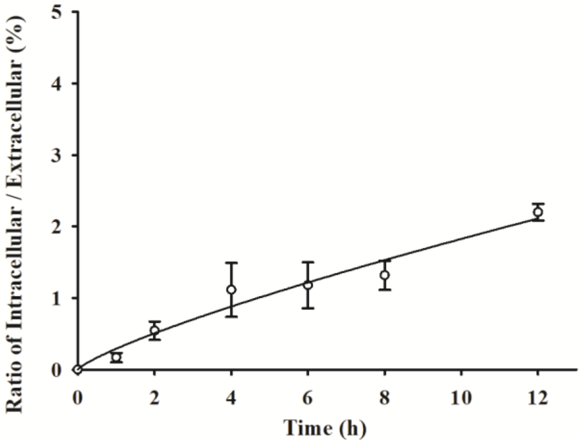

Fig. S5-3-8 Hp1872

Fig. S5-3

Supplementary Material 6

The details of PA effect on the miRNA expression of GES-1 cell.

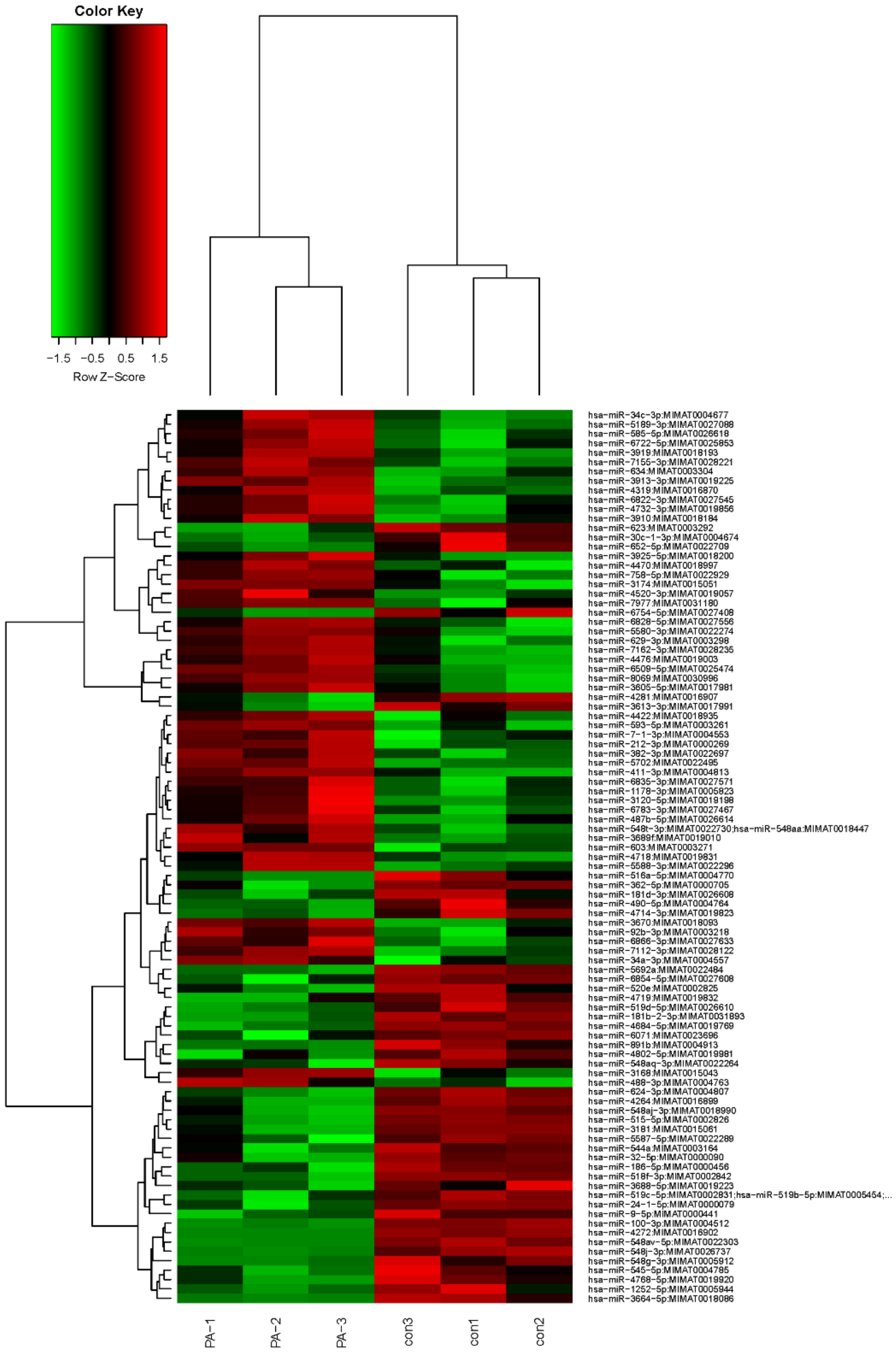

Fig. S6

Supplementary Material 7

Uncropped Western images for Fig. 6. From lane 1 to lane 5, the samples are control, *H. pylori* and PA (12.5, 25 and 50 μM + *H. pylori*), respectively. In pre-experiment, the same sample order is arranged from line 5 to lane 9.

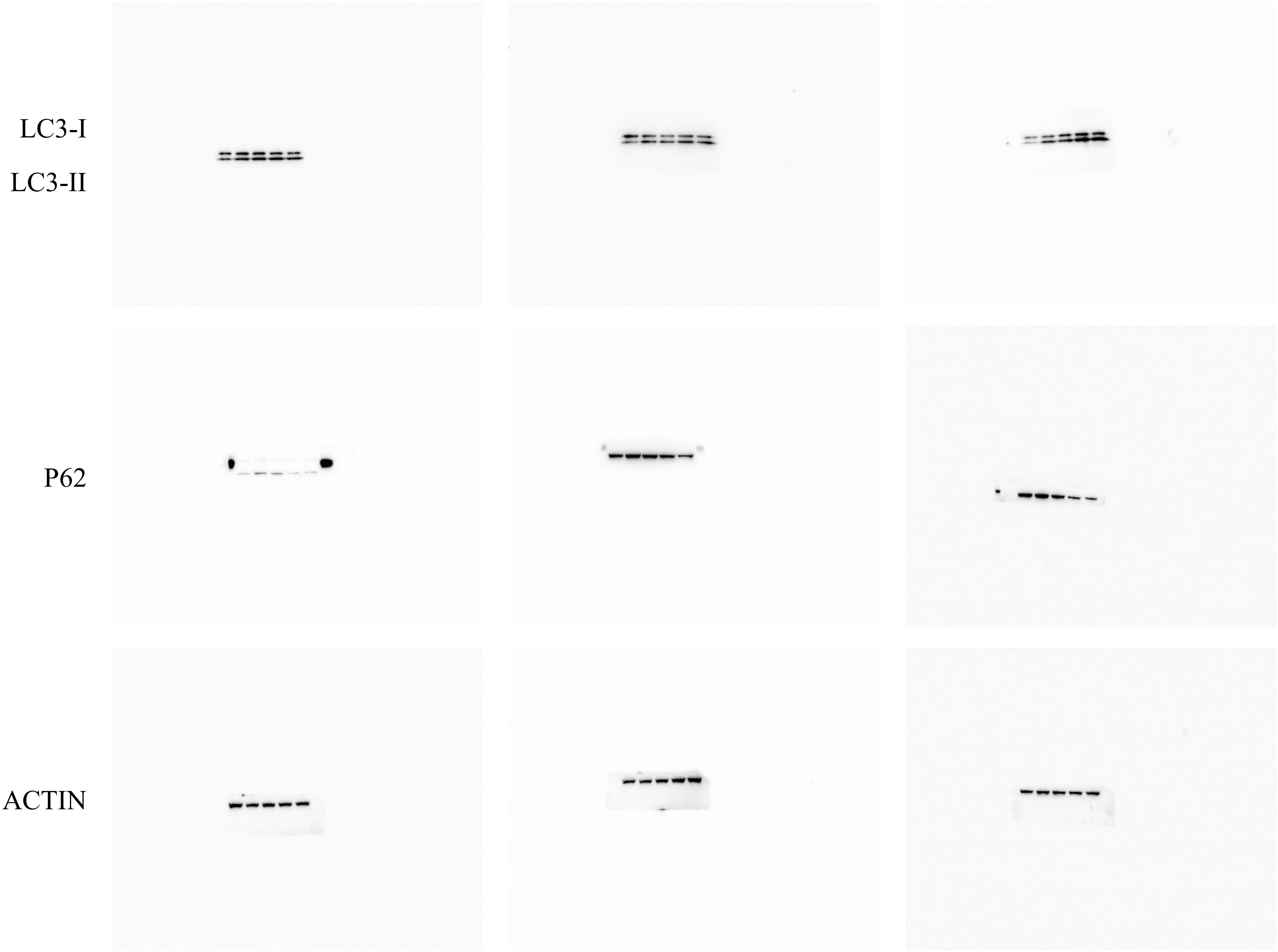

LC3-I
LC3-II
P62
ACTIN

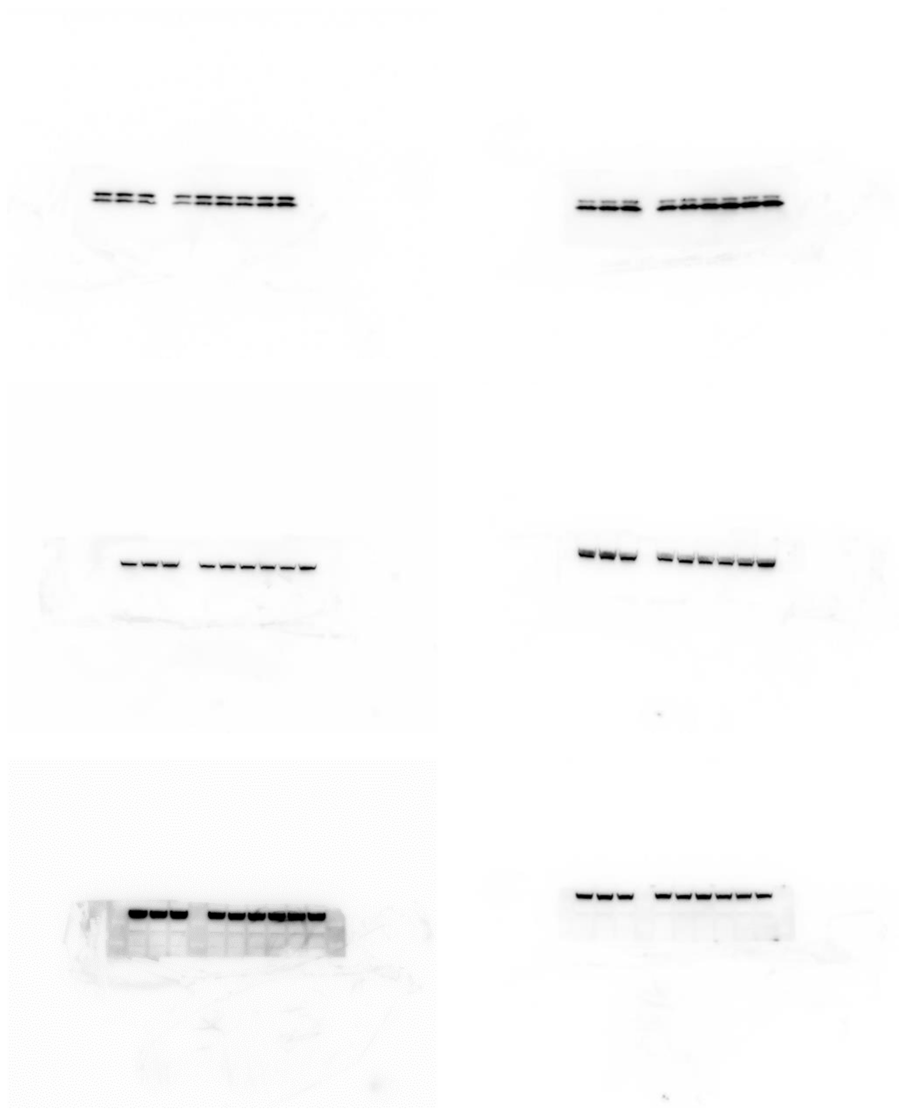

Uncropped Western images for time-dependent TFEB alteration in Fig. 8. For cytoplasmic samples, the samples from lane 2 to lane 5 are treated with *H. pylori* for 0, 6, 12 and 24 h, respectively. For nuclear samples, the same samples were arranged in lane 6 to lane 9 (or lane 2 to lane 5). The sample treated with *H. pylori* for 24 h hasn't been shown in manuscript, because no obvious alteration was observed.

Cytoplasmic

TFEB

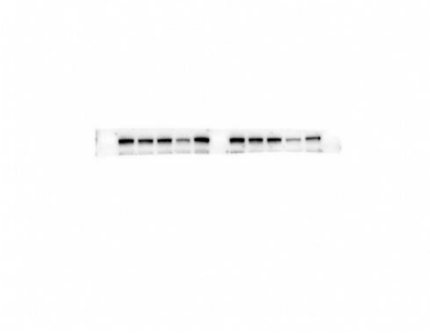

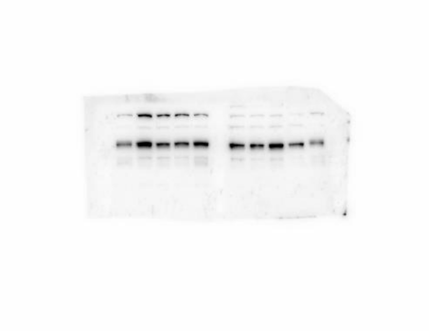

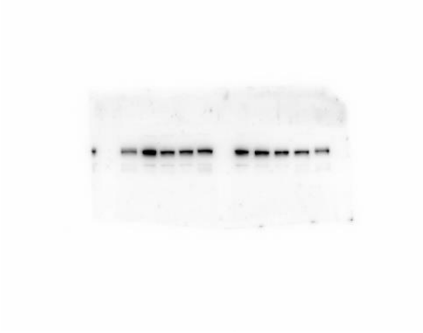

Cytoplasmic

ACTIN

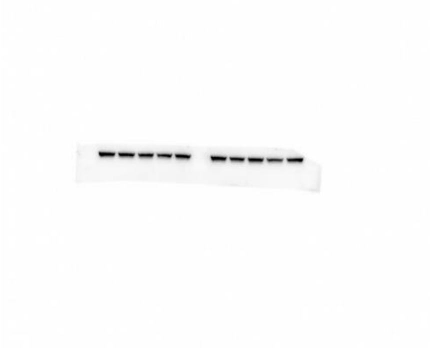

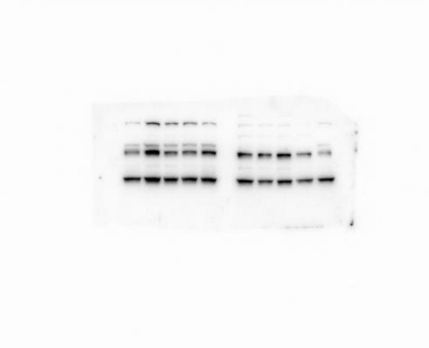

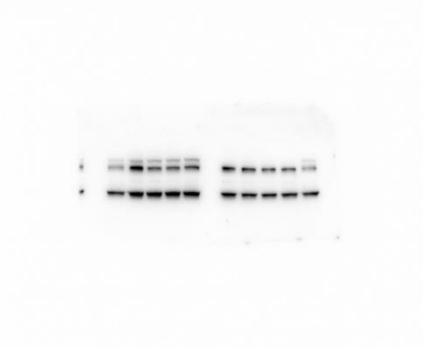

Cytoplasmic

TFEB

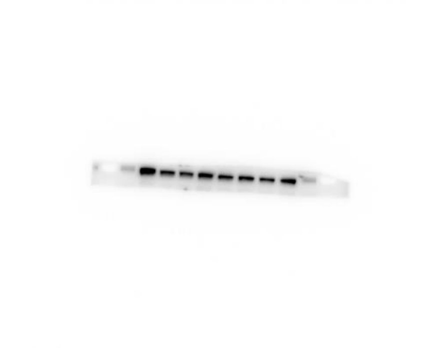

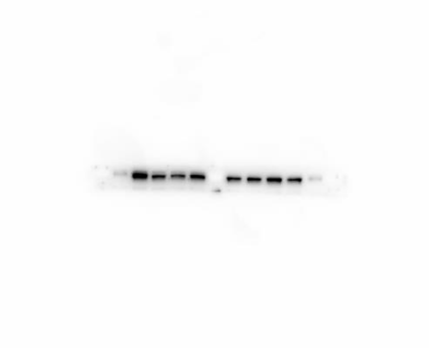

Cytoplasmic

ACTIN

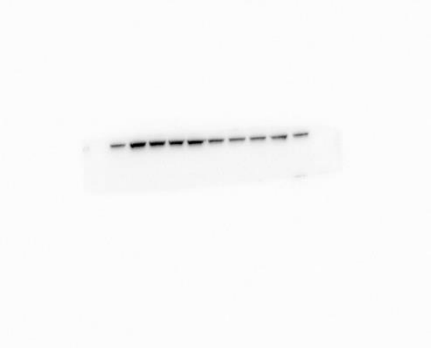

Nuclear
TFEB

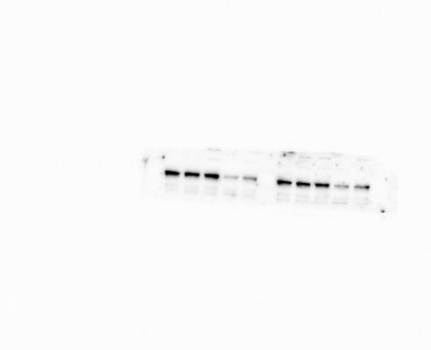
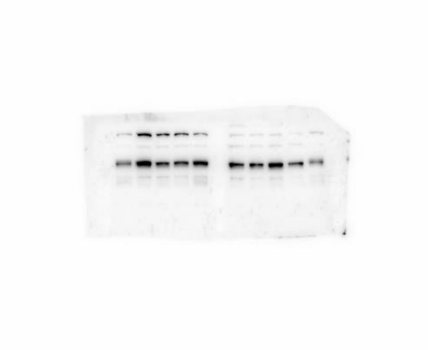
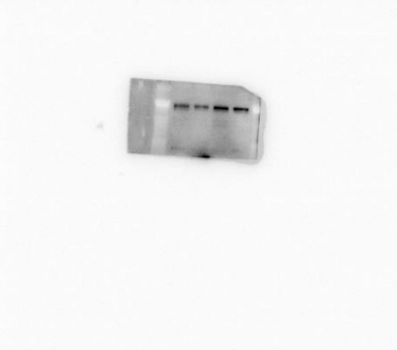

Nuclear
H3

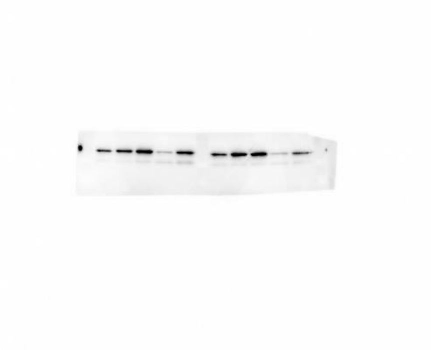
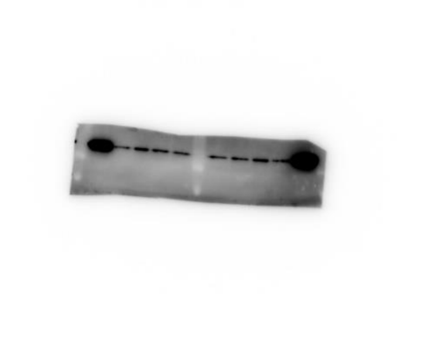
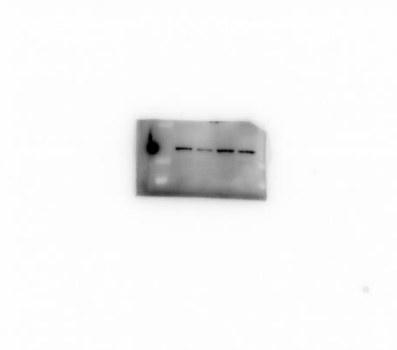

Nuclear
TFEB

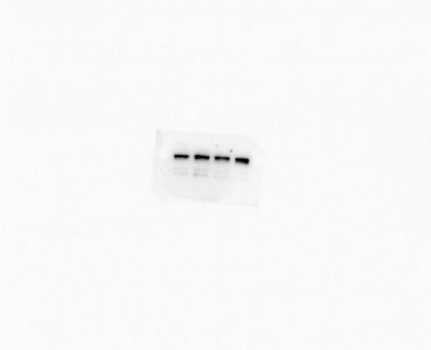
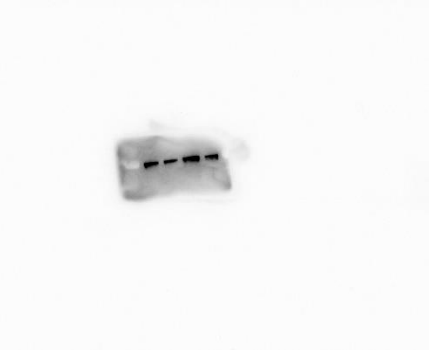

Nuclear
H3

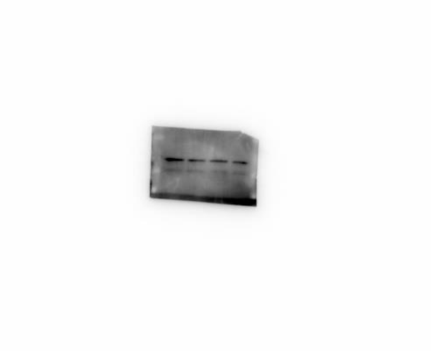
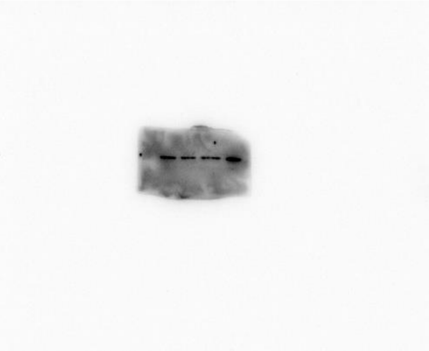

Uncropped Western images for observing the effect of PA on TFEB in Fig. 8. For cytoplasmic samples, the bands from lane 2 to lane 5 are the samples treated with DMSO (control), *H. pylori*, rapamycin+ *H. pylori*, and PA+ *H. pylori*, respectively. For nuclear samples, the same samples were arranged in lane 1 to lane 4.

Cytoplasmic
TFEB

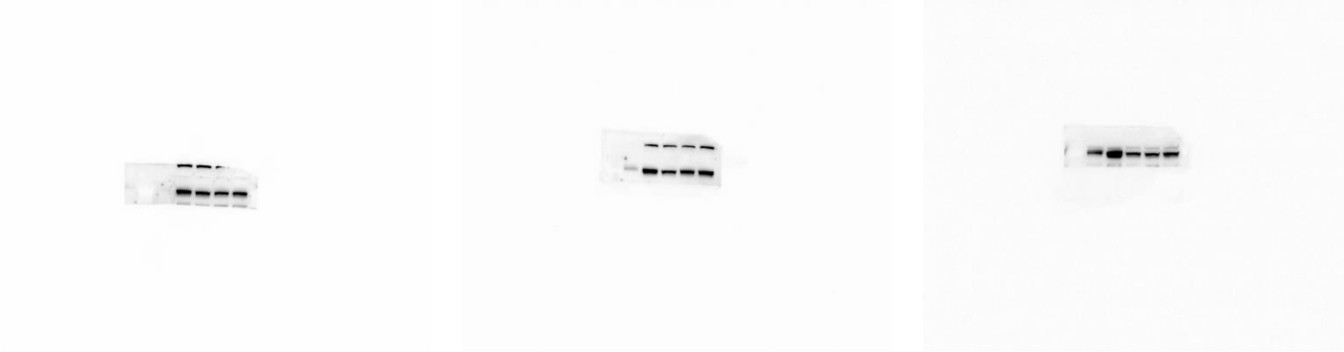

Cytoplasmic
ACTIN

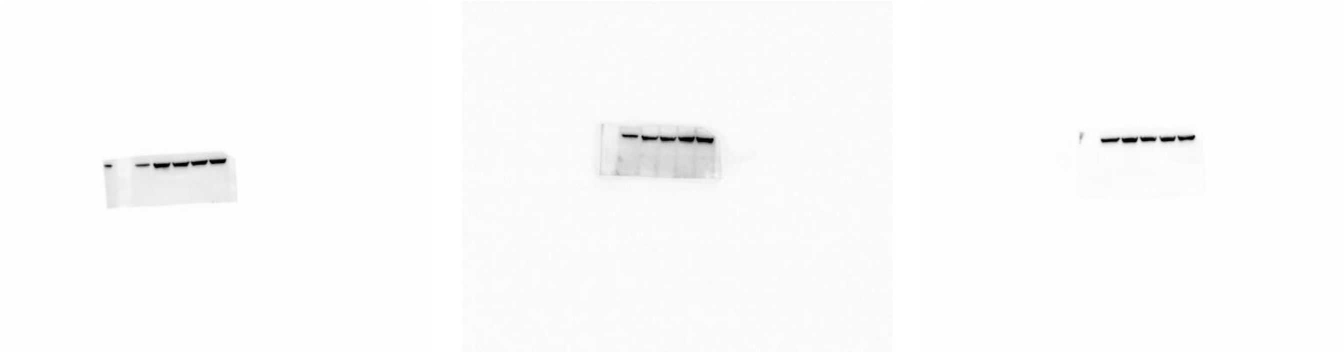

Cytoplasmic
TFEB

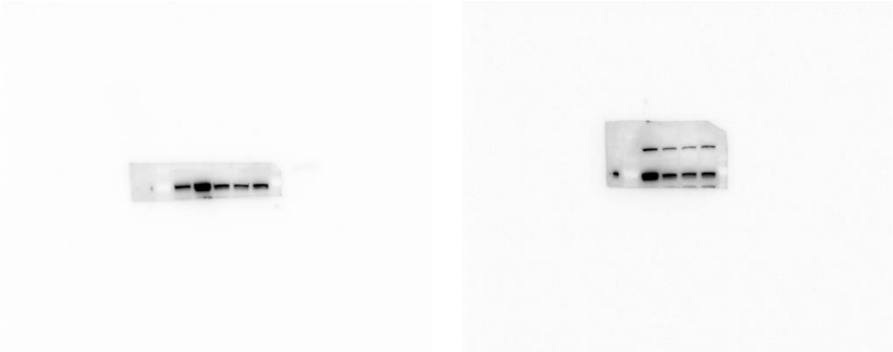

Cytoplasmic
ACTIN

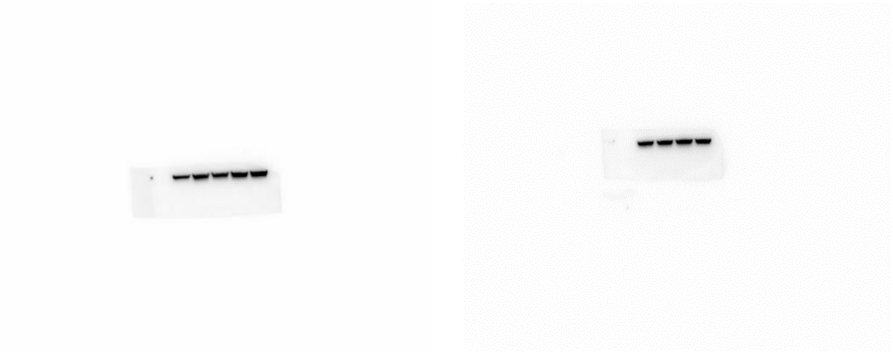

Nuclear TFEB

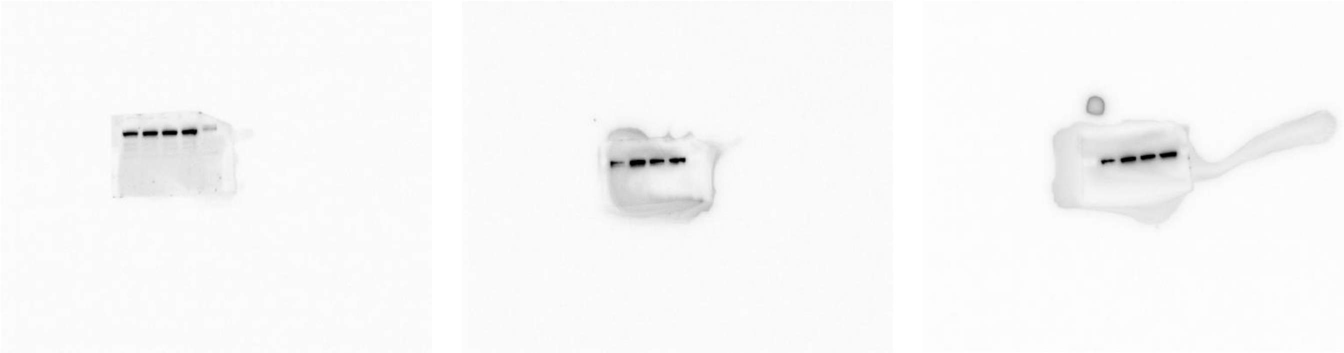

Nuclear H3

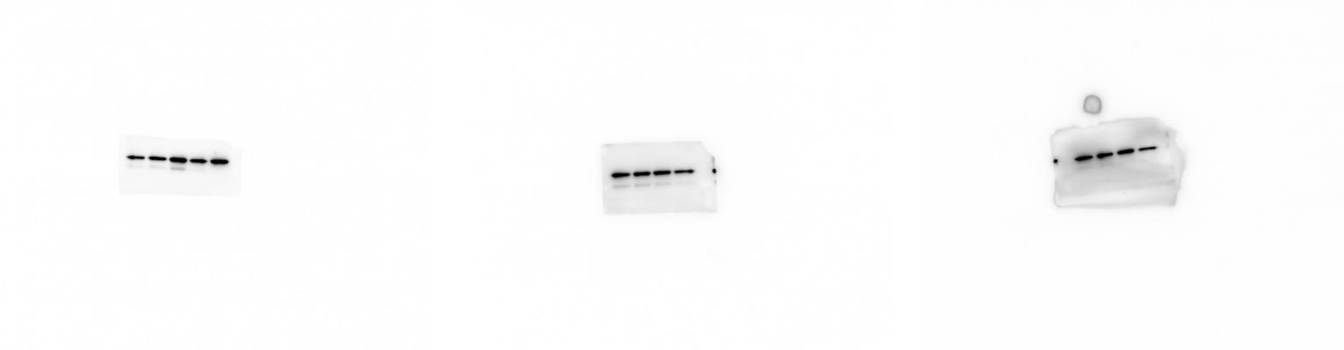

Nuclear TFEB

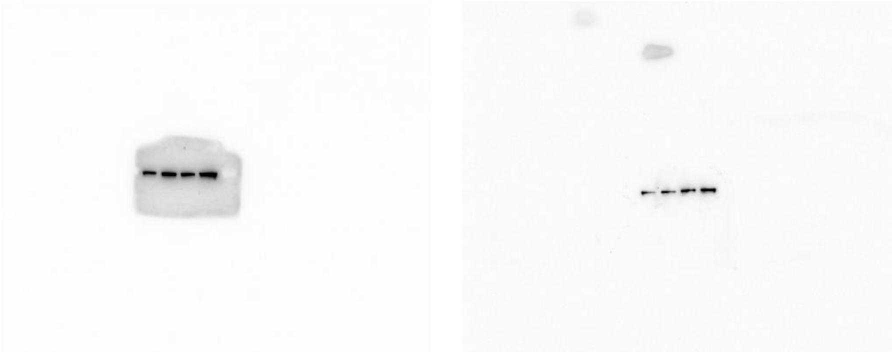

Nuclear H3

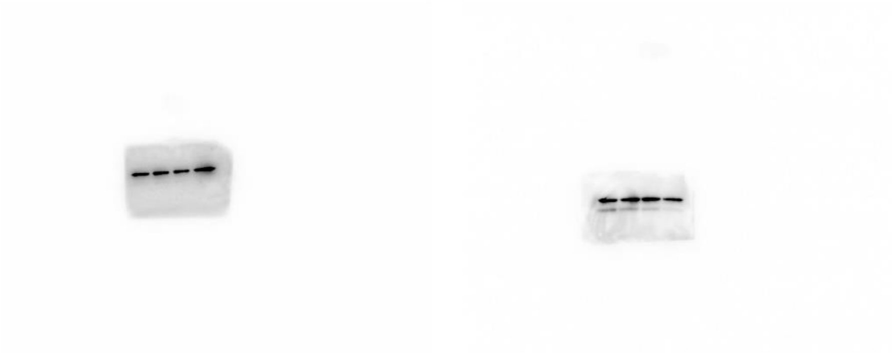

Supplementary Material 8

The virulence genotypes of the standard and four clinical *H. pylori* strains are shown in Table S3. The primers are presented in Table S4.

Table S3 The virulence genotype for *H. pylori* in this research

	vacA	*cagA*	*cagL*	*ureB*	*babA*
NCTC11637	*s1i1m1*	+	+	+	+
NCTC26695	*s1i1m1*	+	+	+	-
SS1	*s2i2m2*	+	+	+	-
Hp1868	*s1i1m1*	+	+	+	+
Hp1869	*s1i1m1*	+	+	+	-
Hp1870	*s1i1m2*	+	+	+	-
Hp1872	*s1i1m2*	+	+	+	+

Table S4 The primers sequence

Gene	Forward primer (5’-3’)	Reverse primer (5’-3’)
vacA s1/2	ATGGAAATACAACAAACACAC	CTGCTTGAATGCGCCAAAC
vacA m1/2	CAATCTGTCCAATCAAGCGAG	GCGTCAAAATAATTCCAAGG
vacA i1	GTTGGGATTGGGGGAATGCCG	TTAATTTAACGCTGTTTGAAG
vacA i2	GTTGGGATTGGGGGAATGCCG	GATCAACGCTCTGATTTGA
cagA	AATACACCAACGCCTCCAAG	TTGTTGCCGCTTTTGCTCTC
cagL	GCAGAATTCATAACAAGCGGCTTAAAG	ATTAGAATTCATAGCCTATCGTCTCAG
ureB	TTCACCCCAACAAATCCCTACAG	ACGGCCCATCGCTTGAGAGT
babA	AATCCAAAAAGGAGAAAAAGTATGAAA	TGTTAGTGATTTCGGTGTAGGACA
